# Creation of haemoglobin A1c direct oxidase from fructosyl peptide oxidase by combined structure-based site specific mutagenesis and random mutagenesis

**DOI:** 10.1038/s41598-018-37806-x

**Published:** 2019-01-30

**Authors:** Noriyuki Ogawa, Takehide Kimura, Fumi Umehara, Yuki Katayama, Go Nagai, Keiko Suzuki, Kazuo Aisaka, Yukie Maruyama, Takafumi Itoh, Wataru Hashimoto, Kousaku Murata, Michio Ichimura

**Affiliations:** 1 Research Laboratories, Kyowa Medex Co., Ltd., 600-1, Minami-ishiki, Nagaizumi-cho, Sunto-gun, Shizuoka 411-0932 Japan; 2 R&D Division, Kyowa Hakko Kirin Co., Ltd., 3-6-6, Asahi-machi, Machida-shi, Tokyo 194-8533 Japan; 3 Division of Food Science and Biotechnology, Graduate School of Agriculture, Kyoto University, Uji, Kyoto 611-0011 Japan; 4 Department of Life Science, Faculty of Science and Engineering, Setsunan University, Neyagawa, Osaka 572-8508 Japan; 5 Faculty of Bioscience and Biotechnology, Fukui Prefectural University, Yoshida-gun, Fukui 911-1195 Japan

## Abstract

The currently available haemoglobin A1c (HbA1c) enzymatic assay consists of two specific steps: proteolysis of HbA1c and oxidation of the liberated fructosyl peptide by fructosyl peptide oxidase (FPOX). To develop a more convenient and high throughput assay, we devised novel protease-free assay system employing modified FPOX with HbA1c oxidation activity, namely HbA1c direct oxidase (HbA1cOX). AnFPOX-15, a modified FPOX from *Aspergillus nidulans*, was selected for conversion to HbA1cOX. As deduced from the crystal structure of AnFPOX-15, R61 was expected to obstruct the entrance of bulky substrates. An R61G mutant was thus constructed to open the gate at the active site. The prepared mutant exhibited significant reactivity for fructosyl hexapeptide (F-6P, N-terminal amino acids of HbA1c), and its crystal structure revealed a wider gate observed for AnFPOX-15. To improve the reactivity for F-6P, several mutagenesis approaches were performed. The ultimately generated AnFPOX-47 exhibited the highest F-6P reactivity and possessed HbA1c oxidation activity. HbA1c levels in blood samples as measured using the direct assay system using AnFPOX-47 were highly correlated with the levels measured using the conventional HPLC method. In this study, FPOX was successfully converted to HbA1cOX, which could represent a novel *in vitro* diagnostic modality for diabetes mellitus.

## Introduction

Haemoglobin A1c (HbA1c) is a species of glycated haemoglobin (Hb) that is generated by non-enzymatic condensation between the amino group of the N-terminal valine of the Hb β-chain and glucose in the red blood cells^1^. This *in vitro* diagnostic modality can serve as a biomarker for diabetes mellitus. HbA1c reflects the blood glucose level over the last 2–3 months^2^, and it is calculated as the percentage of abundance of HbA1c relative to the total Hb concentration. Currently, HbA1c is measured using several assay methods, such as HPLC^3^, the LATEX immunoturbidimetric assay^4^ and enzymatic assay^5^. The recently developed HbA1c enzymatic assay has become particularly popular because of easy handling on conventional autoanalysers and cost-effectiveness in comparison to other assay methods.

**Figure 1:**
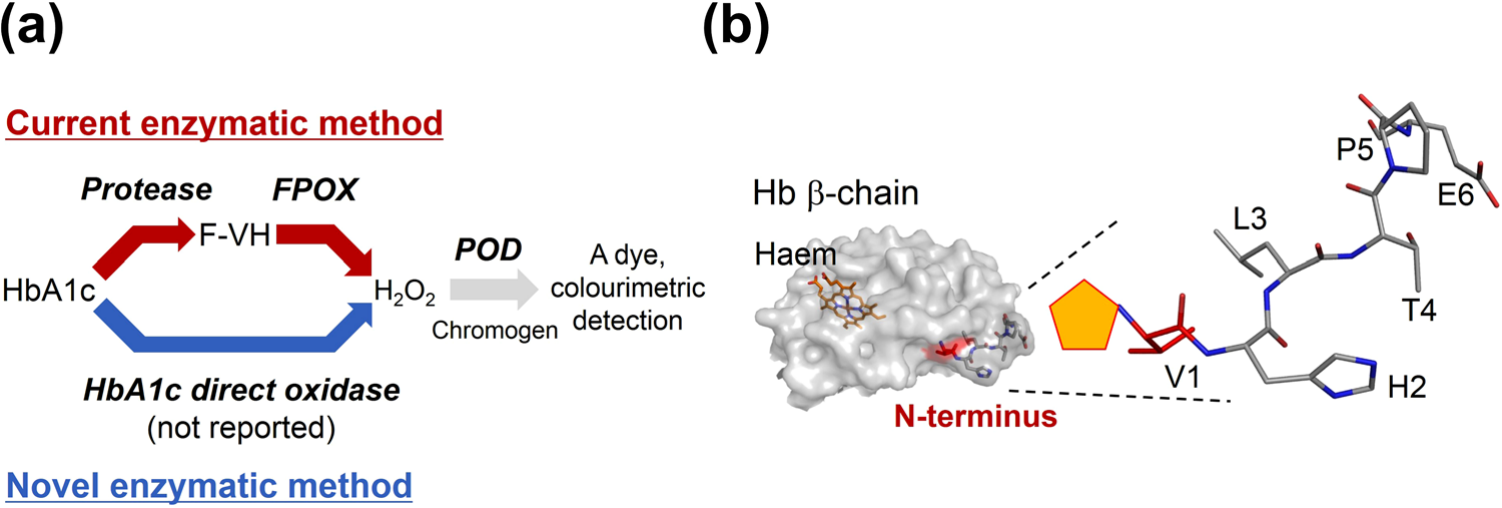
The scheme of the HbA1c enzymatic assay and N-terminal six amino acids of the haemoglobin (Hb) β-chain and its glycation site. (**a**) Reaction scheme of the HbA1c enzymatic method. A red arrow indicates the current enzymatic method consists of specific two tandem enzymatic reaction, namely, proteolysis of HbA1c and the subsequent oxidation of the liberated fructosyl valyl histidine (F-VH) by fructosyl peptide oxidase (FPOX). Afterwards, according to the general detection method, the generated hydrogen peroxide (H_2_O_2_) is then reacted with a chromogen in the presence of peroxidase to produce a dye. The concentration of F-VH can be quantitatively determined by measuring the specific absorption of the dye. The amount of F-VH reflects that of HbA1c stoichiometrically. A blue arrow indicates the novel enzymatic method employing HbA1c direct oxidase (HbA1cOX) to form H_2_O_2_. H_2_O_2_ can be converted to a signal in the same manner as the current method. (**b**) Left, Hb β-chain structure (PDB id: 2ND2) is shown as a surface model, and the haem prosthetic group is shown as an orange stick model. The N-terminal valine, the glycation site of HbA1c, is coloured red. The N-terminal six amino acids are shown as a line model and superimposed on the surface model of Hb β-chain. Right, magnified structure of the N-terminal six amino acids of the Hb β-chain. The orange pentagon at an amino group of the N-terminal valine denotes the fructosyl moiety, suggesting a plausible F-6P structure.

The current HbA1c enzymatic assay consists of two specific enzymatic reactions: i) HbA1c is degenerated by detergents and proteolysed to generate the fructosyl dipeptide fragment, fructosyl valyl histidine (F-VH). “Fructosyl” indicates the attachment of a 1-deoxy-fructosyl moiety to the amino group of the amino acid via glycation. ii) The liberated F-VH reacts with FPOX to generate glucosone, valyl histidine (VH) and hydrogen peroxide (H_2_O_2_). Afterwards, according to the general detection method, the generated H_2_O_2_ is then reacted with a chromogen in the presence of peroxidase to produce a dye. The concentration of F-VH can be quantitatively determined by measuring the specific absorption of the dye. The amount of F-VH reflects that of HbA1c stoichiometrically (Fig. 1(a)).

A number of enzymes, termed fructosyl amino acid oxidase (FAOX), have reactivity for fructosyl amino acids as major substrates^6^. Some of these enzymes, which also exhibit reactivity for fructosyl dipeptides as F-VH are specifically termed fructosyl peptide oxidase (FPOX). Because of their reactivity for F-VH, current HbA1c enzymatic assays often employ FPOX for a more specific measurement of HbA1c^6^. In this report, we termed these fructosyl substrate reactive enzymes FPOX generically.

To meet the rapidly growing demand for more convenient monitoring of diabetes mellitus, we devised to develop protease-free enzymatic HbA1c *in vitro* diagnostics, namely “HbA1c direct enzymatic assay”. This new assay is expected to bring several benefits. In particular, i) excluding the proteolysis step directly contributes to the convenience of measurement, which could also reduce reagent costs. Second, ii) skipping the proteolysis step also opens the possibility of shortening measuring time, which is advantageous for high throughput measurement. To construct such a novel system, we took the strategy to modify FPOX with direct oxidation activity for a whole HbA1c protein.

To date, structurally modified FPOX with improved specificity for HbA1c-derived fragments, such as α-fructosyl valine (F-V) or F-VH, and lower reactivity for ε-fructosyl lysine (ε-F-K), have been developed via several mutagenesis approaches^7–11^. With respect to current assay system, the combination of a modified specific FPOX and adequate protease selection at the first step realises substantially specific measurement of HbA1c.

On the other hand, the idea for creating HbA1cOX via FPOX modification and application to a novel enzymatic assay is referred in some reports^6,12–15^. Some wild-type or modified FPOX have been reported to have oxidation activities for fructosyl hexapeptide (F-6P, Fru-VHLTPE) of the N-terminal amino acids of HbA1c^16^ (Fig. 1(b)). The other modified FPOX were reported to exhibit reactivity for bulky substrates such as fructosyl poly-lysine or fructosyl adamantanamine^12,13^. Despite such active modification studies, no modified FPOX was reported to demonstrate the oxidation activity towards whole glycated proteins, including HbA1c.

We sought to create HbA1cOX by modifying fungal FPOX. First, we tried to isolate template FPOX for modification from a fungal library based on substrate specificity (Supplementary Information). Previous research proposed that fungal FPOX enzymes could be divided into three groups (group I-III) according to sequential identity and substrate preference^6^. Among them, preferential reactivity for α-fructosyl molecules such as F-V and F-VH and low reactivity for ε-fructosyl molecule as ε-F-K is a characteristic of group I FPOX. HbA1c is generated via α-glycation of N-terminal amino group of Hb β-chain. Therefore, group I FPOX were expected to represent favourable templates for creating HbA1cOX. Thus, we performed activity-based screening using a fungal library to obtain a template enzyme, referring to the F-V/ε−F-K ratio as an index. *Aspergillus nidulans* FPOX (AnFPOX-1), which had a remarkably high F-V/ε-F-K ratio, was identified and recombinantly expressed using *Escherichia coli*. Preliminary modification of AnFPOX-1 was performed via cumulative random mutagenesis to improve its reactivity for F-VH as the model substrate of HbA1c and thermal stability. AnFPOX-15, which featured 11 amino acid replacements in AnFPOX-1 exhibited significant F-VH oxidation activity, whereas reactivity for longer fructosyl peptides such as fructosyl tripeptide (F-3P, Fru-VHL), fructosyl tetrapeptide (F-4P, Fru-VHLT) and F-6P was never observed. By contrast, AnFPOX-15 features a robust backbone with high thermal stability (Supplementary Information). We decided to adopt AnFPOX-15 for further drastic structural modification to create HbA1cOX.

To obtain a novel enzyme with beneficial enzymatic characteristics, rational mutagenesis based on the crystal structure is an effective approach. At the beginning of our study, the crystal structure of only one fungal FPOX, namely AfFAOX-II (FPOX group III), was available^14^. Low amino acid sequence identity (37%) and sequential alignment between AfFAOX-II and AnFPOX-15 (Fig. S1 and Table S3) suggested structural differences in active sites and the tunnel shape of active site. Therefore, for detailed and rational design using AnFPOX-15 as a template, its crystal structure was essential information. The target sites for mutagenesis were determined based on the crystal structure, and F-6P was used as the substrate instead of HbA1c to facilitate screening of the enzymes with higher activity than AnFPOX-15.

By combining structure-based site specific mutagenesis and random mutagenesis, we finally generated AnFPOX-47 with dramatically improved F-6P reactivity by replacing 10 amino acids in AnFPOX-15. In particular, replacement of R61 with a smaller amino acid was a key mutation step for obtaining oxidation activity for bulky fructosyl substrates. Using AnFPOX-47, we examined direct oxidation of HbA1c using an assay system without protease and detected signals with high correlation with the values obtained using conventional HPLC method.

In this study, we drastically changed the substrate specificity of FPOX via structure-based site specific mutagenesis and random mutagenesis to create a novel HbA1cOX that can recognise the whole HbA1c protein as a substrate, thus demonstrating the development of a novel concept of HbA1c measurement, “HbA1c direct enzymatic assay”.

## Results and Discussion

### Crystal structure of AnFPOX-15

Through our screening, we identified wild-type AnFPOX-1 produced by *A*. *nidulans*. Random mutagenesis was applied to AnFPOX-1 in a stepwise manner to generate AnFPOX-15. AnFPOX-15 possessed high substrate specificity for F-VH and high thermal stability (Table S1). However, no obtained mutants exhibited reactivity for fructosyl peptides longer than F-VH in the course of AnFPOX-15 generation via random mutagenesis (Supplementary Information). Therefore, we initially attempted to determine the crystal structure of AnFPOX-15 for detailed rational design.

The crystal structure of AnFPOX-15 was resolved using single-wavelength anomalous diffraction (SAD), and both of the crystal structure of the ligand-free AnFPOX-15 and that of fructosyl thioacetate (FSA, mimic of fructosyl glycine, competitive inhibitor of FPOX^14^) complex (here after described as AnFPOX-15/FSA) were determined at 2.60 and 1.85 Å resolution, respectively. Table S4 summarises the results of X-ray data collection and structure refinement. The refined models comprised 431 residues for a protein molecule in an asymmetric unit, although the enzyme consisted of 438 residues. All amino acid residues (P3-R433), except for the N-terminal residues (M1 and A2), could be assigned to the 2*Fo*-*Fc* map; however, the electron-density map for residues S297-I303 and H309-P313 were relatively ambiguous.

**Figure 2:**
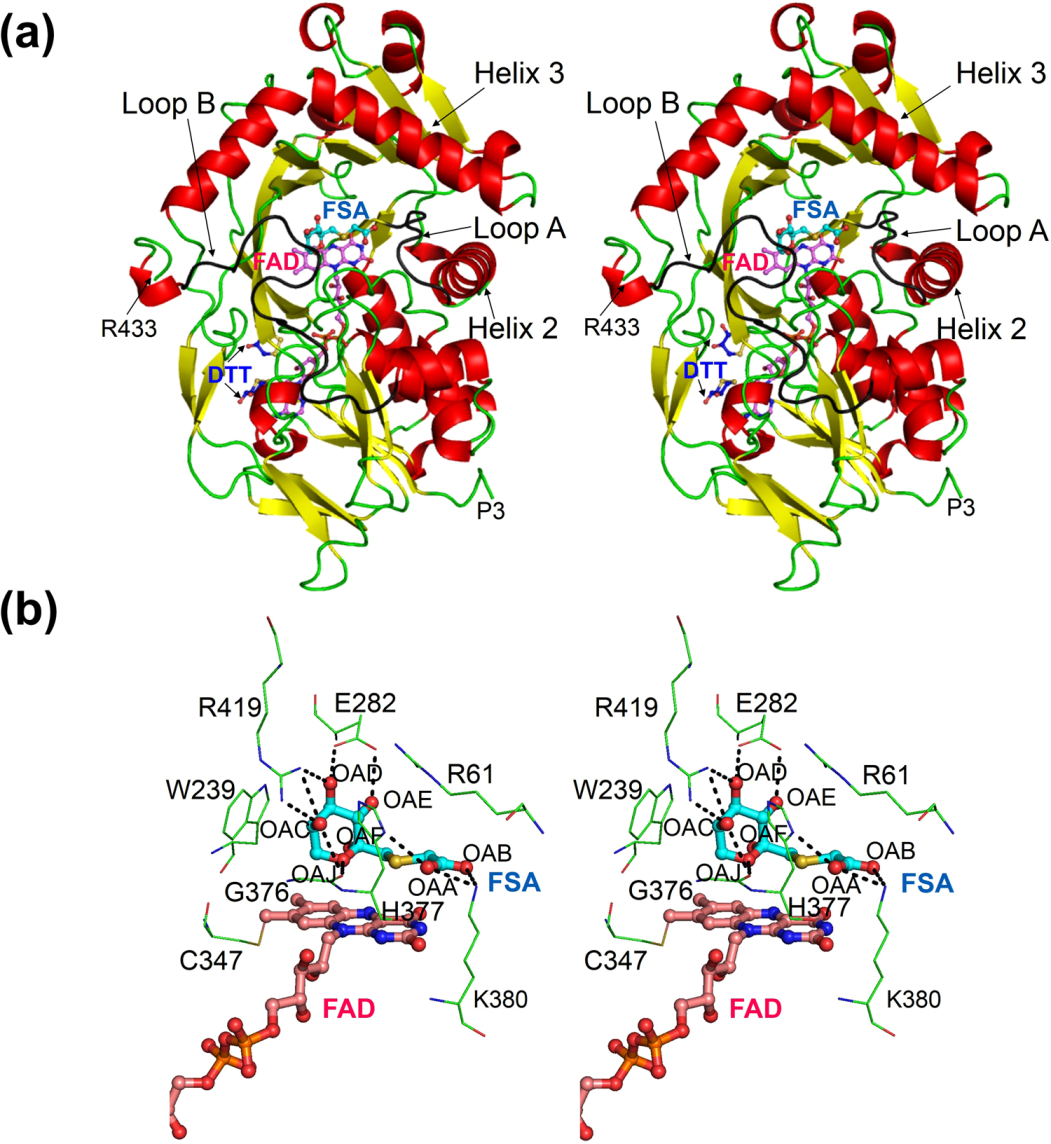
Crystal structure of AnFPOX-15. (**a**) The overall structure of FPOX-15/FSA. Colours denote the secondary structure elements (red: α-helices; yellow: β-strands; green; loops and coils). Loop A and B are shown in gray. FAD, FSA, sulphate ion and oxidised DTT are shown as ball and stick model in cyan, purple and blue respectively. The figures were prepared using cross-eyed stereo diagram. (**b**) The substrate-binding site of FPOX-15/FSA. FSA and the surrounding amino acid residues are shown at cross-eyed stereo diagram. Dashed lines indicate hydrogen bonds between FSA and FPOX-15. Oxygen atoms of FSA molecule are labeled with OAA-OAJ. FAD is also shown partially.

The overall structure of AnFPOX-15 consisted of two domains (Fig. 2(a)): an oxidoreductase FAD-binding domain with the topology of an incomplete anti-parallel β-barrel (two β-sheets with eight β-strands) and four α-helices at one side of the β-barrel; and a catalytic domain with two β-sheets (eight or two β-strands), and two long α-helices (helix 2 and 3) that locate at each side of the sheets, and some short helices. The structure of AnFPOX-15 resembled the known structures of other FPOX enzymes (Table S3). Recently, the crystal structures of group I FPOX, namely *Eupenicillium terrenum* FPOX (EtFPOX)^17^ and *Phaeosphaeria nodorum* FPOX (PnFPOX)^18^, were reported. They both shared high amino acid sequence identity with AnFPOX-15 (Fig. S1 and Table S3) as well as showed quite low rmsd value compared with AnFPOX-15 (0.80 Å and 1.12 Å, respectively), suggesting their structural similarity.

No significant structural difference was observed between the ligand-free AnFPOX-15 and AnFPOX-15/FSA (rmsd = 0.46 Å for 429 *C*_α_ atoms). No gate-closing structural change was observed upon binding of FSA at the active site, which corresponded with the structural characteristics of group I FPOX^17^. Hereafter, unless otherwise indicated, the structural descriptions are provided for the AnFPOX-15/FSA structure.

Interactions between FAD or FSA and AnFPOX-15 are summarised in Fig. 2(b) and Table S5. The flavin ring of FAD and thioacetate group of FSA were arranged in a parallel manner, as firstly reported for the FPOX structure of *A*. *fumigatus* FAOX-II (AfFAOX-II)^14^. The conformations of the amino acid residues surrounding FAD in AnFPOX-15 were essentially common to those in other known FPOX. FSA consists of two moieties, namely fructosyl and thioacetate moieties. The interactions of the fructosyl moiety of FSA with E282, G376 and R419 in AnFPOX-15 were common to those of AfFAOX-II, although the residues that interacted with the thioacetate moiety of FSA varied, suggesting that the substrate preference of FPOX family enzymes was determined by the residues at this site.

Regarding random mutagenesis for AnFPOX-15 generation, introducing the S71Y mutation into AnFPOX-1 led to significant F-VH oxidation activity (Table S1 in Supplemental Information). Kim *et al*. reported that G60 of PnFPOX is the decisive residue for F-VH oxidation activity^19^. Seventy-first residue and 59 residue (corresponding to G60 of PnFPOX) of AnFPOX-15 were structurally distant, as 71 residue is located in helix 2 and behind loop A, whereas 59 residue is located in loop A. They could both contribute to F-VH oxidation activity by affecting the flexibility of loop A.

### Structure-based mutation design

**Figure 3:**
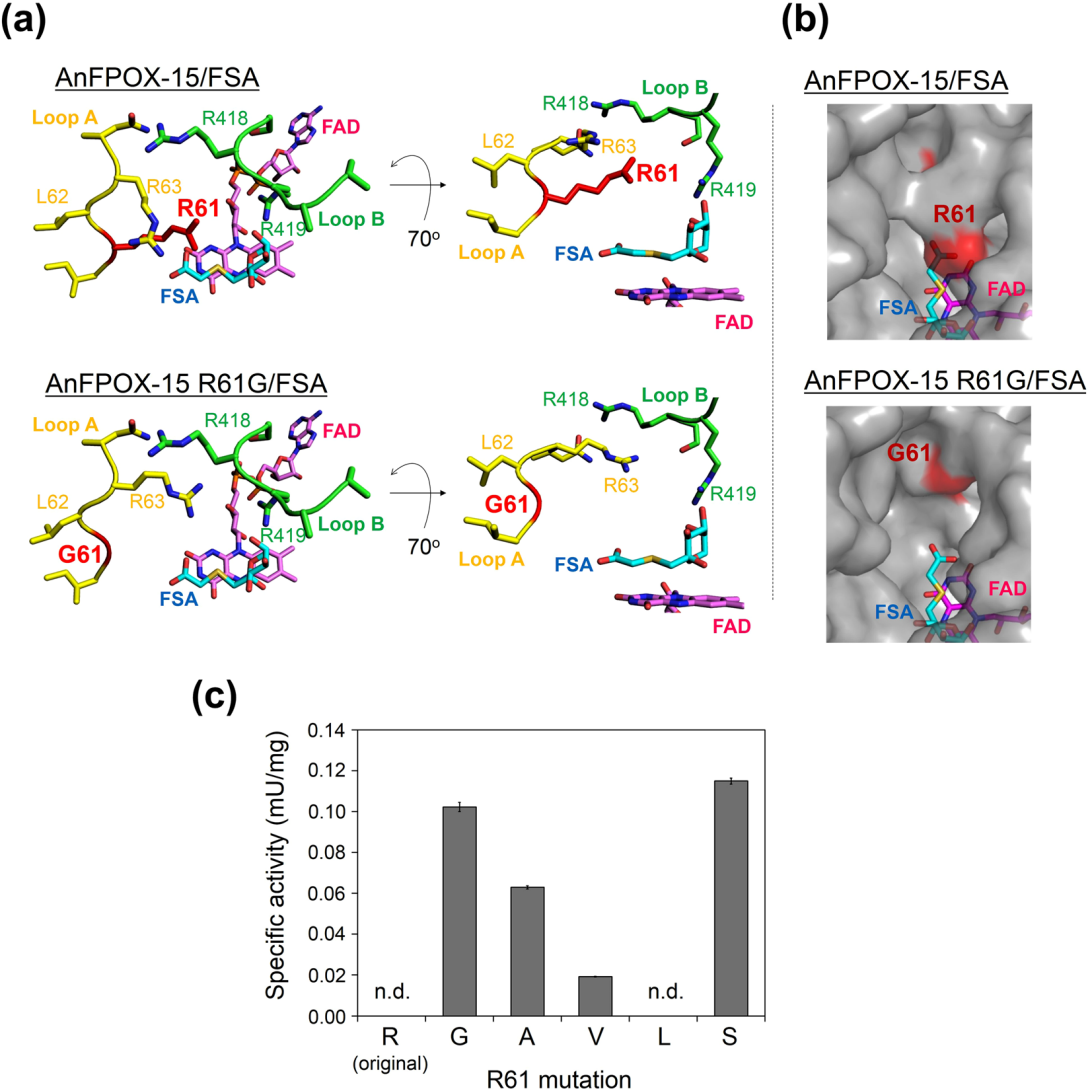
The structure of the active site gates of AnFPOX-15 and AnFPOX-15 R61G and the mutation effect of the R61 residue for F-6P reactivity. (**a**) Coordination of amino acids around the active site of AnFPOX-15. AnFPOX-15/FSA (upper) and AnFPOX-15 R61G/FSA (lower) are shown. The molecule in the right panel is rotated 70° from that in the left panel. R61 residue of AnFPOX-15 and G61 residue of AnFPOX-15 R61G are coloured red. (**b**) Focused molecular surface view of the active site gates of AnFPOX-15/FSA and AnFPOX-15 R61G/FSA. Each 61 residue is shown in red. Coordinated FSA and FAD are shown as stick models in cyan and magenta, respectively. (**c**) Change in F-6P oxidation specific activity by replacing R61 of AnFPOX-15. n.d.: not detected.

The gate for the substrate-binding site was formed by loop A (I60-N64) and loop B (W407-D425) (Fig. 2(a)). As the structure model (Fig. 3(a)upper) shows, R61 and R63 in loop A and R419 in loop B formed a narrow space for the gate. R419 appeared essential for the recognition of fructosyl moiety (Fig. 2(b) and Table S5). Whereas, R61 was located near an amino acid residue of the substrate rather than the fructosyl moiety (Fig. 2(b)). The side-chain of R61 did not form hydrogen bond or salt bridge with FSA; nevertheless, the distance between the *C*_α_ atom of R61 and the oxygen atom (OAB) of FSA was close (5.1 Å) (Fig. 2(b)). That suggests R61 residue does not participate in substrate binding but obstruct entrance of substrate sterically.

Thus, mutation of R61 to smaller residues was expected to widen the space of the substrate gate and improve the affinity between the enzyme and substrates consisting of longer peptides. Based on these observations, we selected R61 as the primary candidate to obtain FPOX with reactivity for whole HbA1c. For the modification procedure, we employed F-6P, a glycated N-terminal six amino acids of HbA1c, as an index substrate for the convenient evaluation instead of HbA1c itself.

The first-generation mutants, in which R61 was replaced with glycine or alanine, displayed significant oxidation activities for F-6P (Fig. 3(c)). To confirm the structural influence of the R61G mutation, the crystal structure of AnFPOX-15 R61G was determined. Ligand-free AnFPOX-15 R61G and AnFPOX-15 R61G/FSA structures were crystallised under similar condition as those used for AnFPOX-15, and the structures were determined via molecular replacement using coordinates of AnFPOX-15 as the initial model. The substrate gate in AnFPOX-15/R61G was apparently wider than that in the original AnFPOX-15 (Fig. 3(a,b)), indicating that the mutation was successfully introduced as designed. No other significant structural differences were found between AnFPOX-15 and AnFPOX-15 R61G (rmsd = 0.148 Å for 431 *C*_α_ atoms). As well as in the case of AnFPOX-15, no significant structural difference was observed between the ligand-free AnFPOX-15 R61G and AnFPOX-15 R61G/FSA (rmsd = 0.17 Å for 431 *C*_α_ atoms).

Site-saturation mutagenesis of R61 revealed that R61S was the best substitution for F-6P oxidation activity (Fig. 3(c)), suggesting that reactivity for F-6P could be induced not only by reducing the size of the side-chain on at 61 residue but by other factors. AnFPOX-15 R61 mutants including R61G, R61A, R61V and R61S were the first AnFPOX mutants with significant F-6P oxidation activities in this study.

Wild-type PnFPOX was reported to have F-6P oxidation activity, although the critical residues in the PnFPOX amino acid sequence for this activity were not specified. Alignment analysis revealed that PnFPOX possesses a serine at the corresponding site of AnFPOX R61 (Fig. S1), suggesting this serine would contribute to F-6P reactivity^16^.

### Structural modification via site specific mutagenesis and random mutagenesis

Using AnFPOX-15 R61S as a template, the neighbouring L62 and R63, which are both aligned in loop A, were also subjected to site-saturation mutagenesis. Consequently, we identified the effective substitutions L62G and R63A. AnFPOX-18 featuring these mutations exhibited 3.49-fold higher F-6P specific activity (0.401 mU/mg; Table 1).

**Table 1:** Summary of AnFPOX mutants generated in this study.

Mutants	Mutations (Number)	F-6P specific activity (mU/mg)	Fold*
AnFPOX-15	none	n.d.	—
AnFPOX-15 R61G	**R61G** (1)	0.102 ± 0.002	—
AnFPOX-15 R61S	**R61S** (1)	0.115 ± 0.001	1.00
AnFPOX-18	R61S/**L62G**/**R63A** (3)	0.401 ± 0.016	3.49
AnFPOX-21	R61S/L62G/R63A/**Y71S**/**L75F**/**M108K**/**D115R** (7)	23.0 ± 0.320	200
AnFPOX-36	R61S/L62G/R63A/Y71S/L75F/D115R/**K108R**/**P66H** (8)	43.0 ± 1.09	374
AnFPOX-47	R61S/L62G/R63A/P66H/Y71S/L75F/M108R/D115R/**F342V**/**A355S** (10)	82.4 ± 2.53	717

Mutations given in bold indicate additional mutations introduced in the generation. Specific activity data are presented as the mean ± SD (n = 3).

*The fold change of F-6P specific activity compared with the value for AnFPOX-15 R61S. The value for AnFPOX-15 R61S was regarded as 1.00. n.d.: not detected.

Next, we attempted the three rounds of random mutagenesis to identify additional effective sites (Fig. S2). To evaluate random mutagenesis library, we employed a two-step activity assay. In the first rough selection, F-V oxidation activity was assayed, and clones exhibiting higher signals (higher rank 25%) were selected. Then, the selected clones were evaluated using F-6P as a substrate, and the clones displaying significant signals were isolated and subjected to sequence analysis. The amino acid position of each degenerated mutation was regarded as an effective site, and they were optimised via site-saturation mutagenesis.

The first round focused on the active site, including loop A and helix 3 (F58-G119), using an AnFPOX-18-expressing plasmid as a template, resulting in the isolation of four effective replacements. Namely, Y71 in helix 2 located behind loop A in AnFPOX-15 structure was replaced with serine. M108 and D115, which were aligned in helix 3, were replaced with lysine and arginine, respectively. Substitution of L75 with phenylalanine also contributed to the elevation of F-6P oxidation activity. AnFPOX-21, which was generated by introducing aforementioned mutations into AnFPOX-18, resulted in significant elevation of F-6P oxidation activity (23.0 mU/mg; Table 1).

Following a second round focused on the M1-G119 region using an AnFPOX-21-expressing plasmid as a template, two additional effective mutations were identified: P66H and K108R. The amino acid at 108 residue was switched from methionine to lysine in a previous random mutagenesis. Mutations in AnFPOX-21 resulted in the generation of AnFPOX-36 mutant with F-6P oxidation activity of 43.0 mU/mg. In the third round using an AnFPOX-36-expressing plasmid as a template, we selected a region that was unlikely to directly participate in substrate-binding (N330-E391). Consequently, two additional mutations, F342V and A355S, were identified. Concerning the two identified mutations, F342V was located away from the active site, and A355S was located on the opposite edge of the active site of helix 3. AnFPOX-47 featuring these two mutations exhibited the highest F-6P oxidation activity (82.4 mU/mg; Table 1).

The F-6P oxidation activities of AnFPOX mutants were dramatically improved via three rounds of random mutagenesis, and AnFPOX-47 displayed 717-fold higher activity than AnFPOX-15 R61S mutant (Table 1).

### Enzymatic characterisation of the generated AnFPOX mutant

**Figure 4:**
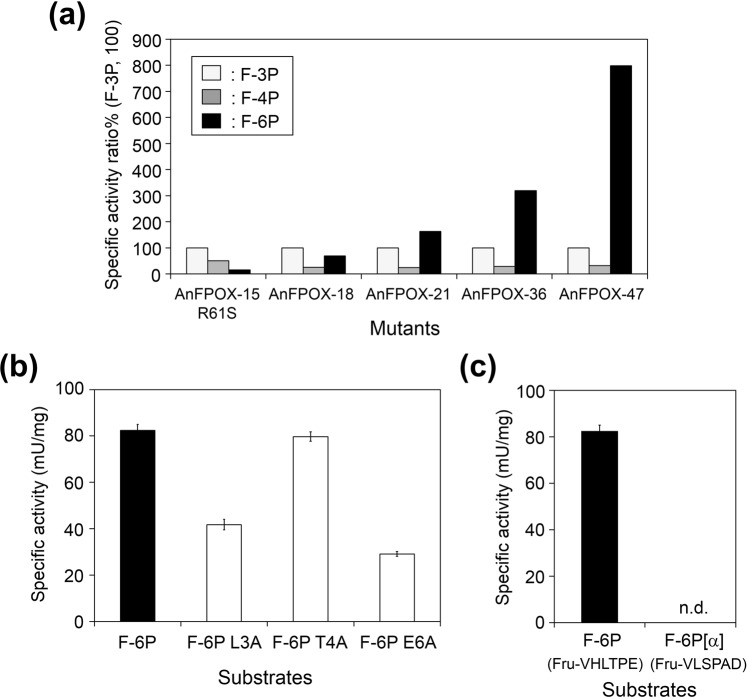
Substrate specificity of the generated AnFPOX mutants. (**a**) Specific activity ratio% of AnFPOX mutants for fructosyl tripeptide (F-3P), fructosyl tetrapeptide (F-4P) and F-6P, each specific activity for F-3P was regarded as 100%. (**b**) Specific activity of AnFPOX-47 for F-6P and its analogues. Data are mean ± SD (n = 3). F-6P analogues share the common amino acid sequence excluding the replacement of one amino acid with alanine. (**c**) Comparison of specific activity for F-6P and F-6P[α]. Data are mean ± SD (n = 3). F-6P[α] is a fructosyl hexapeptide of the glycated Hb α-chain, featuring the amino acid sequence of VLSPAD with glycation at an amino group of the N-terminal valine. F-6P[α] is an interfering substance in the HbA1c measurement. n.d.: not detected.

AnFPOX-47 was evaluated for substrate specificity, especially for fructosyl peptides. AnFPOX-47 exhibited the highest reactivity for F-6P, followed by F-3P and F-4P (Fig. 4(a)). AnFPOX-15 R61S demonstrated substrate preference for shorter fructosyl peptide as F-3P, suggesting that the entrance of a longer substrate was structurally hindered. On the contrary, the subsequently generated mutants clearly exhibited greater preference for F-6P (Fig. 4(a)). The result of the oxidation activity assay of AnFPOX-47 using the alanine replacing analogues of F-6P indicated that the specific recognition for both the L3 and E6 residues of F-6P contributes to high F-6P reactivity of AnFPOX-47 (Fig. 4(b)). No signal was observed in the case of using fructosyl hexapeptide of the glycated Hb α-chain (F-6P[α], Fru-VLSPAD), indicating extremely high specificity for the sequence and structure of F-6P (Fru-VHLTPE), and a low risk of reacting with non-HbA1c glycated Hb via AnFPOX-47 (Fig. 4(c)).

In the application of modified enzymes as diagnostic reagents, their thermal stability is also a critical factor because the stability directly reflects the longevity of the reagent. Following the modification, the thermal stability of AnFPOX-47 decreased from AnFPOX-15, but retained a relatively high level as compared with the wild-type AnFPOX-1 (Fig. S3). The high structural robustness of AnFPOX-15 might contribute greatly to the retention of high stability even after several rounds of mutagenesis.

### Structural assessments of AnFPOX-15 and AnFPOX-21 mutants

To assess structural differences between AnFPOX-15 and AnFPOX-21, for which the largest improvement of F-6P oxidation activity was observed, the obtained X-ray crystal data were structurally analysed.

**Figure 5:**
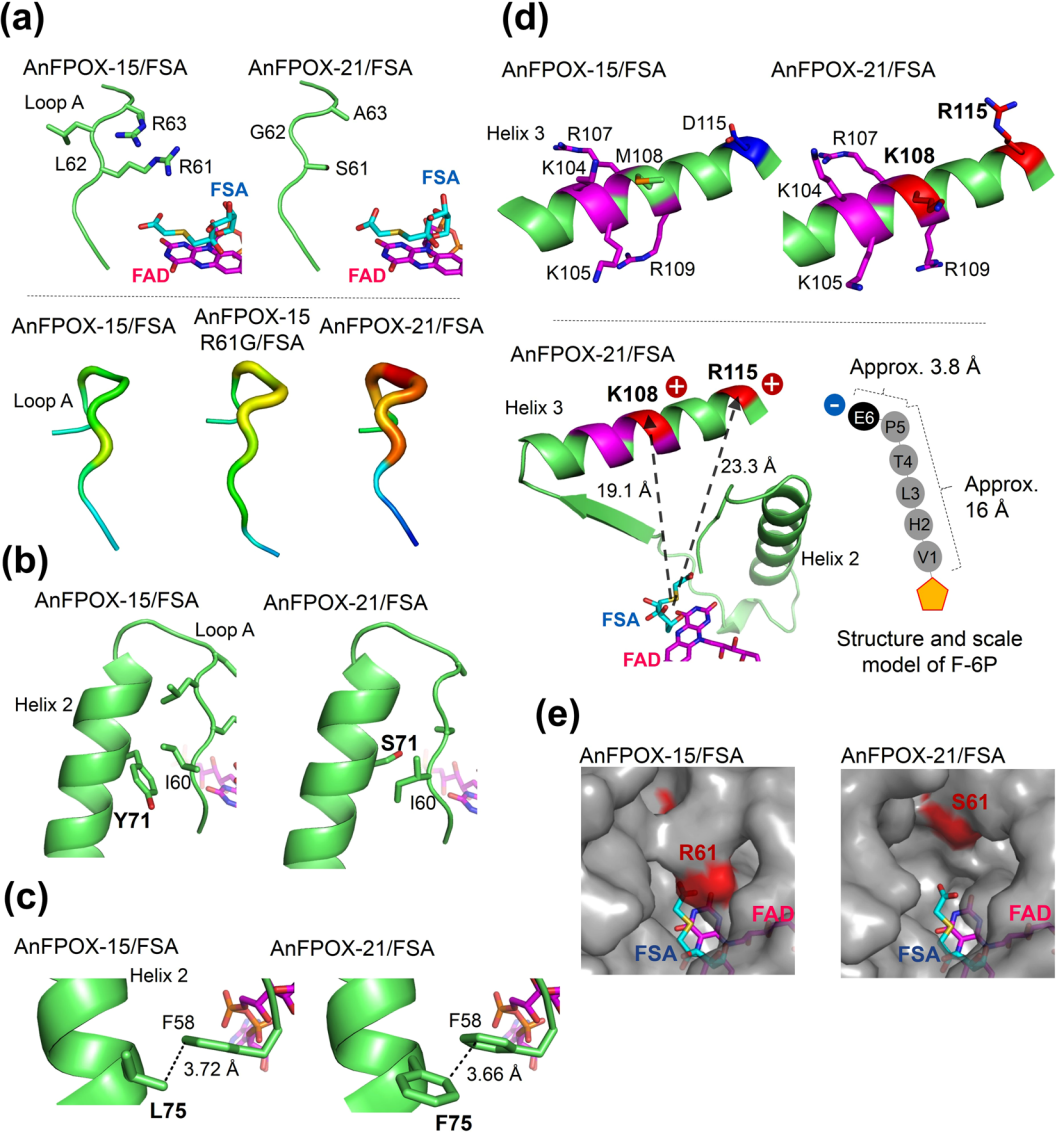
Structural change by mutations introduced into AnFPOX. (**a**) Upper: Structural difference of loop A of AnFPOX-15/FSA and AnFPOX-21/FSA shown as a cartoon model with side-chain as a stick model. Lower: each loop A is shown as setting by *B*-factor putty on PyMOL programme. Warm colour shows high *B*-factor, and the reduced *B*-factor following in the order of red, orange, yellow, sky blue then blue. (**b**) Structural difference of 71 residue in helix 2 was compared between AnFPOX-15/FSA and AnFPOX-21/FSA. Y71 residue was replaced with serine in AnFPOX-21. (**c**) Structural difference of 75 residue in helix 2 was shown as a cartoon model to compare 75 residue between AnFPOX-15/FSA and AnFPOX-21/FSA. L75 residue was replaced with phenylalanine in AnFPOX-21. (**d**) The upper panel shows structural difference of helix 3 between AnFPOX-15/FSA and AnFPOX-21/FSA. Each structure is shown as a cartoon model. The side-chain of the featuring residues is shown as a stick model. Magenta indicates innate basic residues on AnFPOX-15, blue indicates acidic residues and red indicates basic residues newly introduced in the generation of AnFPOX-21. Lower panel, the left figure shows the active site of AnFPOX-21/FSA. Dotted arrows show the distance from the sulphur atom of FSA to the α-carbon of each K108 and R115. Right figure shows rough structural model of F-6P. An orange pentagon indicates a fructosyl moiety. The distances were measured using the crystal structure of the haemoglobin β-chain (PDB id: 2DN2) using the PyMOL programme. The distance of approx. 16 Å is a length from nitrogen of N-terminal valine to α-carbon of P5, and distance of approx. 3.8 Å is a length from α-carbon of P5 to that of E6. The side-chain length of R115 is approximately 7 Å, and that of E6 of F-6P is approximately 4.7 Å. When F-6P is coordinated onto FAD of AnFPOX-21, E6 of F-6P is located around the active site gate, enabling the formation of electrostatic interactions between them. (**e**) The active site gates of AnFPOX-15/FSA and AnFPOX-21/FSA are shown in surface model. Coordinated FSA and FAD are shown as cyan and magenta, respectively.

AnFPOX-21/FSA was crystallised and the structure was determined via molecular replacement. The crystal belonged to *P*6_5_, and it contained two enzymes arranged in an asymmetric unit. Although all amino acid residues from P3 to N434 were identified in one molecule, but S61, G62, and A63 in the other molecule were omitted from the final coordinate file because of the poor electron-density map. No significant structural difference was noted between AnFPOX-15 and AnFPOX-21 (rmsd = 0.436 Å and 0.441 Å for 431 and 427 *C*_α_ atoms, respectively), excluding for the structures in mutated residues, suggesting conservation of the elemental architecture of AnFPOX even after gaining significant F-6P oxidation activity by introducing seven mutations. With respect to AnFPOX-21 the *B*-factor values of the *C*_α_ atom of loop A (S61-N64) was significantly high even in coordinate-determined chain (S61, 94.22 Å^3^; G62, 84.36 Å^3^; A63, 94.43 Å^3^), suggesting structure flexibility of loop A because of the mutations. The flexibility of the main-chain in this region was not observed in AnFPOX-15 and AnFPOX-15 R61G (Fig. 5(a)lower).

As S61, G62 and A63 have a smaller side-chain size in AnFPOX-21 than original AnFPOX-15, the mutations also alleviate structural interference for the entrance of bulky substrates. Therefore, the structural simplicity and flexibility resulting from these mutations are believed to contribute to the observed increased F-6P reactivity (Fig. 5(a)).

In addition, F-6P possesses histidine residue (H2) which could carry positive charge under physiological condition. Therefore, there is a possibility that cancellation of charge repulsion between R61 and R63 with H2 of F-6P via the mutations facilitates entrance of F-6P into catalytic site of AnFPOX mutants.

PnFPOX and some of the derivatives were reported to have F-6P oxidation activity^16^. In that report, exchanging loop 1 structure (corresponding to loop A of AnFPOX-15) of CoFPOX (non-F-6P reactive FPOX) with that of PnFPOX (F-6P reactive FPOX) resulted in generation of CoFPOX chimera enzyme with F-6P reactivity^16^. This result also corresponds to our result that modification of loop A of AnFPOX was elementally important for acquiring F-6P reactivity.

The four mutations identified on the first round of random mutagenesis, namely Y71S, L75F, M108K and D115R, significantly contribute to the improvement of F-6P oxidation activity (57-fold improvement compared with that of AnFPOX-18). The contribution of these four mutations could be divided into two aspects: i) indirect effects on the flexibility of loop A (Y71S and L75F) and ii) conversion of the electric charges in helix 3 (M108K and D115R).

Mutation of Y71 in the AnFPOX-21 mutant to serine reduced the size of the side-chain. Considering the entrance of bulky substrates into the active site, loop A in particular would be pushed backwards to accommodate the substrate. The Y71S mutation could permit the acceptance of such a structural change by alleviating structural interference with the facing I60 residue (Fig. 5(b)). Our study revealed that substitution of S71 in AnFPOX-1 with tyrosine conferred F-VH reactivity (Table S1). The obtained mutation after the AnFPOX-15 mutagenesis study demonstrated that the ancestral amino acid (serine) was effective for F-6P reactivity, implying that the beneficial structure for F-VH oxidation activity is not necessarily in concordance with that for F-6P oxidation activity.

L75F, which is located in helix 2 and behind loop A, also contributes to the enhancement of F-6P oxidation activity, even though no significant structural change was induced. The L75F mutation probably had a fine-tuning effect on the interaction between loop A and helix 2 (Fig. 5(c)).

M108K and D115R, which are located in helix 3, resulted in alignment of six positive-charged amino acids in a condensed manner (Fig. 5(d)upper). When F-6P enters the active site and its fructosyl moiety is coordinated on FAD, E6 (sixth glutamic acid of F-6P) is estimated to be placed near the gate of the active site (Fig. 5(d)lower). The result of the activity assay using the F-6P analogues suggests the importance of E6; therefore, electrostatic interactions between E6 and the aligned positive-charged amino acids in helix 3 would contribute to the stabilisation of F-6P binding.

K104 and K105 were previously introduced in the generation of AnFPOX-15 from glutamic acid and glycine, respectively. These basic amino acids could also support the electrostatic interaction with the E6 of F-6P; hence, using AnFPOX-15 as a modification template may have proven advantageous for our purpose (Fig. 5(d)upper).

An activity assay using F-6P analogues also suggested the importance of L3 of F-6P (Fig. 4(b)). Considering the distance from the fructosyl moiety of F-6P, the hydrophobic side-chain of A63, switched from arginine residues, would play a role in recognition.

The surface model of the AnFPOX-21/FSA structure illustrated the opened active site gate even after FSA binding (Fig. 5(e)). No gate-closing structural change upon substrate (inhibitor) binding is a specific characteristic of group I FPOX^17^, which is also observed for AnFPOX-15/FSA and AnFPOX-15 R61G/FSA structures. In addition, the active site gate of AnFPOX-21 is apparently wider than that of AnFPOX-15, and the thioacetate moiety of FSA coordinated on AnFPOX-21 was observable from outside the molecule (Fig. 5(e)). This wide-opened gate of AnFPOX-21 is expected to be advantageous for F-6P accessibility and reactivity because the larger space should permit longer peptides to be accommodated.

As the crystal structures of mutants generated after AnFPOX-21 have not been solved, the effects of replacement identified in the second and third rounds of random mutagenesis were considered using the structural models generated using the PyMOL programme based on the AnFPOX-21/FSA structure.

In the second round of random mutagenesis, two effective replacements were identified, namely P66H and K108R, although their contribution to F-6P oxidation activity was relatively modest (1.87-fold increase versus AnFPOX-21).

P66 residue is placed on the connexion of loop A and helix 2 (Fig. S4). As shown by the increased *B*-factor of loop A in AnFPOX-21 (Fig. 5(a)), high flexibility of loopA was expected to be beneficial for F-6P reactivity. Judging from the structural model of the P66H mutation generated by the PyMOL programme, the P66H mutation is also expected to effect on flexibility of loop A.

Replacement of K108 with arginine results in elongation of the positive-charged side-chain, from approximately 5.4 Å of that of lysine to approximately 7 Å of that of arginine. The elongated side-chain could widen the supporting range of electrostatic interaction with the E6 of F-6P.

In the third round of random mutagenesis, F342V and A355S were identified as mutations that elevate F-6P oxidation activity (1.92-fold growth compared with AnFPOX-36), although their contributions are still unclear.

F342 in AnFPOX-21 is located completely away from the active site (Fig. S5(a)). In the mutation study, replacement of F342 in AnFPOX-36 with valine resulted in slight elevation of F-6P reactivity and reduction of F-V reactivity. Conversely, replacement of F342 with glycine reduced both F-V and F-6P reactivities (Fig. S5(b)). Shimasaki *et al*. reported that N56 of PnFPOX (corresponding to conserved N55 residue of AnFPOX), which is located on the *re*-face side of the isoalloxasine ring of FAD of PnFPOX, similarly as F342 of AnFPOX, plays an important role in O_2_ invasion, and it is indispensable for the oxidase reaction^18^. Taken together, the F342 mutation may also influence the oxidative half-reaction of FPOX.

A355S is positioned on the opposite side of the active cleft from helix 3 (Fig. S6). Although the role of this site remains unclear, S355 is believed to facilitate the entrance of F-6P into the active site.

### Direct oxidation of HbA1c by AnFPOX mutants

In the assay using Hb as a sample, strong absorption of Hb interfered with detection of the colourimetric reaction signals. Hence, to evaluate direct oxidation activity for glycated Hb, we prepared ApoHb from human Hb, from which the haem prosthetic group was removed, for convenient evaluation. Following the method of preparing Apo myoglobin^20^, ApoHb was successfully prepared as indicated by the presence of two major bands corresponding to the α- and β-chains of Hb-protein on SDS-PAGE (Fig. S7).

**Figure 6:**
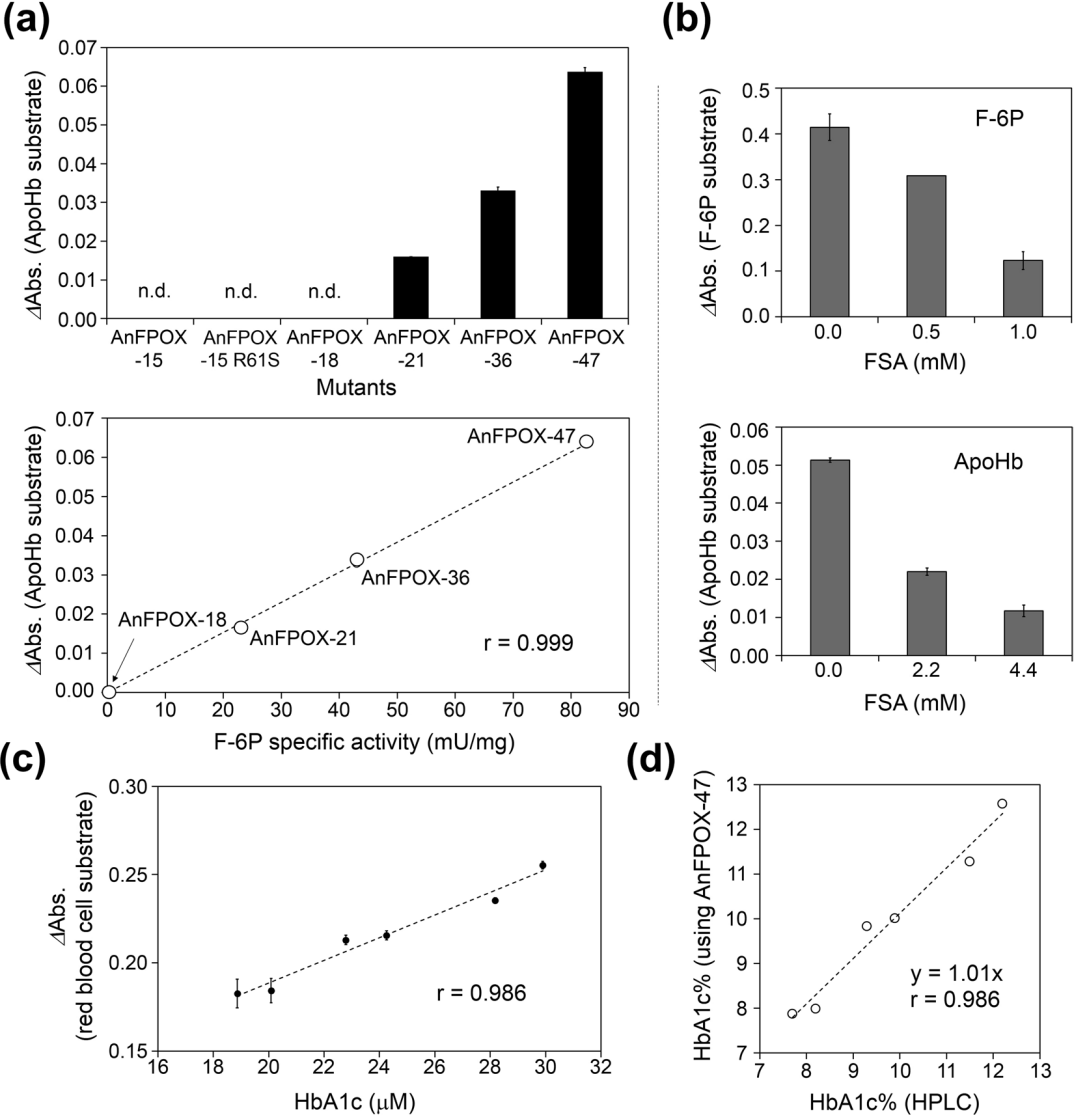
Evaluation of the HbA1c direct oxidation activity of the generated mutants. (**a**) Upper panel, the detection of ApoHb oxidation activity using each of the generated AnFPOX mutants (final concentration of 230 μg/mL) and ApoHb (final concentration of 1.36 mg/mL, including 7.1% HbA1c). A signal was calculated from the change in absorbance during incubation at 37 °C for 1 h by subtracting absorbance change in reagent without enzymes. Data are presented as the mean ± SD (n = 3). n.d.: not detected. Lower panel, correlation between the reaction signal intensity using ApoHb (final concentration of 1.36 mg/mL, including 7.1% HbA1c) as a substrate and the F-6P specific activity (mU/mg) of each of the mutants. Data are the mean of triplicate measurement. (**b**) Inhibitor (FSA) susceptibility assay of AnFPOX-47 using F-6P (final concentration of 0.1 mM) and ApoHb (final concentration of 1.36 mg/mL, including 7.1% HbA1c). AnFPOX-47 was used at final concentrations of 2.3 and 217 μg/mL for F-6P and ApoHb, respectively. FSA was added and mixed prior to substrate addition. Data are presented as the mean ± SD (n = 3). (**c**) Correlation between the signals (*Δ*Abs) obtained via the reaction of AnFPOX-47 with concentration of HbA1c included in Hb samples. Data are presented as the mean ± SD (n = 4). The concentrations of HbA1c was calculated from HbA1c% determined by HPLC and the total Hb concentration determined using Hb-SLS method^21^. AnFPOX-47 (final concentration of 2.6 mg/mL) was subjected to reaction. The signal (*Δ*Abs.) was calculated from the change in absorbance during incubation for 1 h at 37 °C. (**d**) Correlation between the HPLC method and our developed assay system using AnFPOX-47. HbA1c% was calculated from the average of measured value obtained with AnFPOX-47 based on a calibration curve prepared with two independently measured Hb samples (HbA1c%: 6.7 and 12.3).

Using ApoHb as a substrate, we evaluated the direct oxidation activity of each of the generated mutants. As Fig. 6(a)upper shows, significant signals were detected for AnFPOX-21 and the later mutants, and the signal intensities were apparently correlated with the F-6P specific activity of each mutant (Fig. 6(a)lower).

To confirm further whether the detected signals actually resulted from the enzymatic reaction of FPOX, the effect of FSA (a competitive inhibitor of FPOX) on oxidation activity was evaluated using AnFPOX-47. The signal for the ApoHb oxidation activity and that for F-6P oxidation activity were clearly inhibited by FSA in a concentration-dependent manner (Fig. 6(b)). Therefore, the detected oxidation activity was enzymatically catalysed by AnFPOX-47.

Finally, we investigated the HbA1c direct oxidation activity of AnFPOX-47 using washed human erythrocytes as a sample to evaluate the practical potential for its diagnostic use. As a significant signals were detected in this assay system without a protease pre-treatment step, it is strongly implied that the signals were derived from direct oxidation of the whole HbA1c protein. Furthermore, signals measured by AnFPOX-47 and the concentrations of HbA1c determined by HPLC method exhibited a high correlation (Fig. 6(c)).

The concentration of HbA1c in each of the samples was calculated based on the calibration curve prepared using washed erythrocyte samples, which included a known concentration of HbA1c. HbA1c% (abundance of HbA1c molecule over total Hb) for each sample was calculated using the measured and calculated concentration of HbA1c and that of Hb determined by Hb-SLS kit (Wako, Japan) of which principle was described by Oshiro *et al*.^21^.The resulting HbA1c% values of the measured samples actually exhibited a high correlation with the values determined by HPLC (Fig. 6(d)), suggesting that AnFPOX-47 could work as the HbA1cOX.

## Conclusion

In this study, we aimed to create HbA1cOX through structural modification of the partially modified fungal FPOX, AnFPOX-15. The crystal structure of AnFPOX-15 illustrated that R61 apparently obstructs the entrance of bulky substrates such as F-6P into the active site. From these observations, the generated R61G mutant acquired significant oxidation activity for F-6P, and the wider space of substrate gate in R61G mutant was confirmed via its crystal structure.

Further mutagenesis using a combination of structure-based site specific mutagenesis and random mutagenesis yielded AnFPOX-47 with most highly elevated F-6P oxidation activity. Biochemical characterisation using erythrocyte samples demonstrated that AnFPOX-47 is a novel HbA1cOX.

From the perspective of protein engineering, we succeeded in drastically converting the substrate specificity from fructosyl peptides to the whole HbA1c protein, which could lead to the development of more convenient and high throughput *in vitro* diagnostics without involving any protease treatment step.

To establish the actual *in vitro* diagnostics for HbA1c using direct enzymatic oxidation system, further optimisation of the assay conditions is indispensable. The development of an “HbA1c direct enzymatic assay” is ongoing.

## Method

### Enzyme expression and purification

*Escherichia coli* DH5α harbouring the pTrc-AnFPOX-15 vector was developed as described in the Supplementary Information. The strain was cultured at 30 °C in Luria-Bertani (LB) medium (50 mg/L Amp.) with shaking. For the selenomethionine-derivative of AnFPOX-15, *E*. *coli* B834 (DE3) harbouring the pTrc-AnFPOX-15 vector was cultured in minimal medium including 25 μg/mL selenomethionine. Protein expression was induced by adding isopropyl-β-D-thiogalactopyranoside (final concentration, 0.1 mM), when the turbidity reached approximately 0.5 at 600 nm, followed by continued culture with shaking 16 °C for 44 h.

The recombinant AnFPOX was purified from the cultured cells as described in the Supplementary Information. In brief, ammonium sulphate precipitation and the subsequent two steps of column chromatography were employed. Purity was confirmed by SDS-PAGE. All AnFPOX mutants could be purified via the same procedure (Fig. S8). Protein contents were determined by measuring absorbance at 280 nm and assuming that *E*_280_ = 1.54 (AnFPOX-15) corresponded to 1 mg/mL^22^. The contents of other mutants were also determined in the same manner using their *E*_280_ values.

### Activity assay of AnFPOX mutants

Fructosyl substrates and the inhibitor (FSA) were synthesised and purchased from Peptide Institute, Inc. (Osaka, Japan)

The activity assay was performed as follows: First, 190 μL of the mixture, containing 10 mM potassium phosphate buffer (pH7.0), 3.5 U/L horseradish peroxidase, 0.5 mM 4-aminoantipyrine, and 0.5 mM N-ethyl-N-(2-hydroxy-3-sulfopropyl)-3-methylaniline, was mixed with 20 μL of 1 mM F-6P, and 10 μL of samples on multi-well plate. After incubation at 30 °C, the change of the absorbance at 550 and 690 nm (reference) was measured using infinite F200 (Tecan, Switzerland). Activity was calculated as the change in absorbance based on the calibration curve prepared on the absorbance derived from known concentrations of H_2_O_2_. One unit of activity was defined as the quantity of enzyme that could catalyse the formation of 1 μmol of H_2_O_2_ per minute. Specific activity was defined as the activity catalysed by 1 mg of FPOX. For high-sensitivity measurement, 30 μM DA-67 (Wako, Japan) was employed and absorbance at 660 and 750 nm (reference) was used for the measurement.

For evaluation on screening, cell-free extracts of clones were prepared on multi-well plates as described in Supplementary Information.

### Crystallisation and X-ray diffraction

For crystallisation of AnFPOX-15, purified AnFPOX-15 selenomethionine-derivative was concentrated to 6.5 mg/mL using the Centriprep (Merck Millipore, USA). In the case of inhibitor complex, fructosyl thioacetate (FSA) was added at 10 mM concentration.

The crystallisation conditions were initially screened by sparse-matrix screening in a 96-well Intelli-plate (Art Robbins instruments) by commercial crystallisation kits from Hampton Research (Crystal Screen 1 and 2), Jena Science (JBScreen Classic 1–10) and Emerald Bio Systems (Wizard Classic Crystallization Screen 1 and 2), at 20 °C using the sitting-drop vapour-diffusion method. The crystals of AnFPOX-15 selenomethionine-derivative and the FSA complex were obtained after incubation for 3–7 days, in Wizard Classic 1 No. 33 (2.0 M ammonium sulphate, 0.2 M lithium sulphate, and 0.1 M *N*-cyclohexyl-3-aminopropanesulfonic acid (CAPS) buffer, pH 10.5. Trigonal crystals were generated under the optimised condition of 1.75 M ammonium sulphate, 0.2 M lithium sulphate, and 0.08 M CAPS buffer (pH 10.4). The crystals of AnFPOX-15 R61G and AnFPOX-15 R61G/FSA were obtained in the similar manner for AnFPOX-15.

For the AnFPOX-21/FSA, purified AnFPOX-21 was concentrated to 20 mg/mL, to which 10 mM of FSA was added. The crystallisation conditions were screened following the method as described above, using the sitting-drop vapour-diffusion method. Crystal of AnFPOX-21/FSA was obtained after incubation for 2 weeks at 20 °C, in PGA Screen-CF-D9 solution containing 20% (w/v) PEG 3350, 0.1 M 2-morpholinoethanesulfonic acid (MES; pH6.5) and 5% (w/v) poly-γ-glutamic acid (molecular mass: 200–400 kDa) in The PGA Screen Kit (Molecular Dimensions, USA).

For cryoprotection, a crystal was soaked in the reservoir solution containing 20% glycerol and picked up with a mounted nylon loop (Hampton Research). The crystal was then placed directly into a nitrogen gas stream at −173 °C. X-ray diffraction images were acquired at −173 °C under a nitrogen gas stream with a Quantum 210 CCD Detector (ADSC) and synchrotron radiation (λ = 0.97980 Å for ligand-free AnFPOX-15 or 1.00000 Å for other crystals) at the BL-38B1 station of SPring-8 (Hyogo, Japan). In the case of the AnFPOX-15/FSA, data were obtained using the MAR225HE CCD detector (Rayonix) at BL-44XU station of SPring-8. X-ray diffraction images of AnFPOX-21/FSA were acquired at −173 °C under a nitrogen gas stream with the Dectris Pilatus3 S 6 M Detector and synchrotron radiation (λ = 0.98000 Å) at the BL17A Station of Photon Factory (Ibaraki, Japan).

### Structure determination and refinement

The diffraction data sets were processed using the HKL2000 programme package (HKL Research, Inc., USA). The crystal structure of AnFPOX-15 was resolved using SAD with the selenomethionine-derivative crystal. Phase determination and initial model building for the AnFPOX-15 derivative were performed using the AutoSol wizard^23^ supplemented with the PHENIX graphical interface^24^. The winCOOT programme (http://bernhardcl.github.io/coot/) was used to manually modify the initial model. Initial rigid body refinement and several rounds of restrained refinement against the dataset were conducted using the phenix.refine programme with the Translation/Libration/Screw (TLS) refinement. Water molecules were automatically incorporated where the *Fo*-*Fc* and *2Fo*-*Fc* electron density map showed a density of > 3.0 and 1.3 σ, respectively. Figures for ribbon plots were prepared using the PyMOL programme (Schrödinger, LLC, New York, USA). The crystal structures of the AnFPOX-15/FSA, AnFPOX-15 R61G, AnFPOX-15 R61G/FSA and AnFPOX-21/FSA were also determined through the molecular replacement method using the Phaser programme^25^ in the PHENIX graphical interface using the structure of ligand-free AnFPOX-15 as a search model. Refined structures were validated using the MolProbity programme^26^ in the PHENIX graphical interface. The coordinates used in this report were obtained from the Protein Data Bank (PDB), Research Collaboratory for Structural Bioinformatics, Rutgers University, New Brunswick, NJ (http://www.rcsb.org/). RMS deviation between the two models was calculated using the SSM superpose function in the winCOOT programme.

### Site specific mutagenesis and site-saturation mutagenesis

Site specific mutagenesis was employed using pTrc-AnFPOX plasmid as a template following the methods described in the QuikChange Site-directed Mutagenesis kit (Stratagene, CA, USA), except that KOD-Plus-polymerase was used. Oligonucleotides were synthesised at the Hokkaido System Science (Japan). Oligonucleotides sequences used in this study were summarised in Table S6. Introduced mutations were confirmed by DNA sequencing by ABI3700 sequencer with two primers, pTrc-F and pTrc-R. *E*. *coli* DH5α cell was transformed using generated plasmid.

Site-saturation mutagenesis was performed following the same method as in site specific mutagenesis, except for using primer pairs containing NNS (N: A, T, G, or C, S: G or C) on the target site. Two hundreds of colonies were evaluated to isolate favourable clones for subsequent analysis.

### Random mutagenesis

Random mutagenesis was performed via error-prone PCR following the method reported by Kawate *et al*.^27^ using rTaq polymerase (Toyobo, Japan). The reaction mixture was prepared following manufacture’s protocol except for using dNTP mixture consisted of 0.2 mM dATP, 1 mM dTTP, 0.1 mM dGTP and 1 mM dCTP. Ten nano gram of plasmid DNA was added as a template. PCR condition was as follows: 94 °C, 2 min, followed by 45 cycles of 94 °C, 30 sec, 50 °C, 30 sec, and 72 °C, 30 sec, then 72 °C, 1 min.

Amplified PCR product was ligated into the template plasmid and introduced into *E*. *coli* DH5α. The colonies grown on LB agar medium (50 mg/L Amp.) were evaluated as a library. In brief, the colonies were picked and their cell free extract was prepared on multi-well plate. In a first round of evaluation, 25% of the clones that exhibited higher signals using F-V (final concentration of 0.09 mM) were selected. In the subsequent round, the selected colonies were then evaluated using F-6P (final concentration of 0.03 mM). The clones that exhibited higher signal were selected. Approximately 5,000 clones were evaluated on a single round of random mutagenesis.

### HbA1cOX activity assay

Human red blood cell specimens with determined HbA1c% values were purchased from Discovery Life Sciences (CA, USA). Their HbA1c% were determined via the conventional HPLC (Variant II Turbo Haemoglobin Testing System, Bio-Rad, CA, USA).

ApoHb was prepared from human red blood cell specimens following the method described by Harrison *et al*.^20^. The obtained ApoHb was dissolved in 5% of Anhitol 20 N solution (lauryldimethylamine oxide; Kao Chemical, Japan).

Concerning HbA1c direct oxidation assay for the ApoHb (7.1% HbA1c) or intact HbA1c, the same assay system used for F-6P was employed. Prior to the reaction, red blood cell specimens were haemolysed by 5% Anhitol 20 N solution and incubated at 37 °C for 15 min. The reaction signals were defined as the change in absorbance during incubation at 37 °C for 1 h. The Hb concentrations of haemolysed samples were determined via Hb-SLS kit^21^ (Wako).

### Accession codes

The atomic coordinates and structure factors of AnFPOX-15 selenomethionine-derivative, AnFPOX-15 selenomethionine-derivative FSA complex, AnFPOX-15 R61G, AnFPOX-15 R61G/FSA and AnFPOX-21/FSA have been deposited in the Protein Data Bank with the accession id of 6A6R, 6A6S, 6A6T, 6A6U and 6A6V, respectively.

## Supplementary information

Dataset 1

## References

[1] Cho SJ, Roman G, Yeboah F, Konishi Y (2007). The road to advanced glycation end products: a mechanistic perspective. Current medicinal chemistry.

[2] Bunn HF, Gabbay KH, Gallop PM (1978). The glycosylation of hemoglobin: relevance to diabetes mellitus. Science (New York, N.Y.).

[3] Jeppsson JO (2002). Approved IFCC reference method for the measurement of HbA1c in human blood. Clinical chemistry and laboratory medicine.

[4] Hamwi A, Schweiger CR, Veitl M, Schmid R (1995). Quantitative measurement of HbA1c by an immunoturbidimetric assay compared to a standard HPLC method. American journal of clinical pathology.

[5] Sakurabayashi I (2003). New enzymatic assay for glycohemoglobin. Clinical chemistry.

[6] Ferri S, Kim S, Tsugawa W, Sode K (2009). Review of fructosyl amino acid oxidase engineering research: a glimpse into the future of hemoglobin A1c biosensing. Journal of diabetes science and technology.

[7] Fujiwara M (2006). Alteration of substrate specificity of fructosyl-amino acid oxidase from *Ulocladium* sp. JS-103. Journal of bioscience and bioengineering.

[8] Miura S, Ferri S, Tsugawa W, Kim S, Sode K (2008). Development of fructosyl amine oxidase specific to fructosyl valine by site-directed mutagenesis. Protein engineering, design & selection: PEDS.

[9] Kim SMS, Ferri S, Tsgawa W, Sode K (2009). Cumulative effect of amino acid substitution for the development of fructosyl valine-specific fructosyl amine oxidase. ENZYME MICROB TECHNOL.

[10] Wu X, Chen SG, Petrash JM, Monnier VM (2002). Alteration of substrate selectivity through mutation of two arginine residues in the binding site of amadoriase II from Aspergillus sp. Biochemistry.

[11] Hirokawa K, Ichiyanagi A, Kajiyama N (2008). Enhancement of thermostability of fungal deglycating enzymes by directed evolution. Applied microbiology and biotechnology.

[12] Qian Y, Zheng J, Lin Z (2013). Loop engineering of amadoriase II and mutational cooperativity. Applied microbiology and biotechnology.

[13] Zheng J, Guan H, Xu L, Yang R, Lin Z (2010). Engineered amadoriase II exhibiting expanded substrate range. Applied microbiology and biotechnology.

[14] Collard F (2008). Crystal structure of the deglycating enzyme fructosamine oxidase (amadoriase II). The Journal of biological chemistry.

[15] Rigoldi F (2016). Crystal structure of the deglycating enzyme Amadoriase I in its free form and substrate-bound complex. Proteins.

[16] Ferri S, Miyamoto Y, Sakaguchi-Mikami A, Tsugawa W, Sode K (2013). Engineering fructosyl peptide oxidase to improve activity toward the fructosyl hexapeptide standard for HbA1c measurement. Molecular biotechnology.

[17] Gan W (2015). Structural basis of the substrate specificity of the FPOD/FAOD family revealed by fructosyl peptide oxidase from *Eupenicillium terrenum*. *Acta crystallographica*. Section F, Structural biology communications.

[18] Shimasaki T, Yoshida H, Kamitori S, Sode K (2017). X-ray structures of fructosyl peptide oxidases revealing residues responsible for gating oxygen access in the oxidative half reaction. Scientific reports.

[19] Kim S, Ferri S, Tsugawa W, Mori K, Sode K (2010). Motif-based search for a novel fructosyl peptide oxidase from genome databases. Biotechnology and bioengineering.

[20] Harrison SC, Blout ER (1965). REVERSIBLE CONFORMATIONAL CHANGES OF MYOGLOBIN AND APOMYOGLOBIN. The Journal of biological chemistry.

[21] Oshiro I, Takenaka T, Maeda J (1982). New method for hemoglobin determination by using sodium lauryl sulfate (SLS). Clinical biochemistry.

[22] Gill SC, von Hippel PH (1989). Calculation of protein extinction coefficients from amino acid sequence data. Analytical biochemistry.

[23] Terwilliger TC (2009). Decision-making in structure solution using Bayesian estimates of map quality: the PHENIX AutoSol wizard. Acta crystallographica. Section D, Biological crystallography.

[24] Echols N (2012). Graphical tools for macromolecular crystallography in PHENIX. Journal of applied crystallography.

[25] McCoy AJ (2007). Phaser crystallographic software. Journal of applied crystallography.

[26] Chen VB (2010). MolProbity: all-atom structure validation for macromolecular crystallography. Acta crystallographica. Section D, Biological crystallography.

[27] Kawate H, Landis DM, Loeb LA (2002). Distribution of mutations in human thymidylate synthase yielding resistance to 5-fluorodeoxyuridine. The Journal of biological chemistry.

