# Supplementary material for: Creation of haemoglobin A1c direct oxidase from fructosyl peptide oxidase by combined structure-based site specific mutagenesis and random mutagenesis
Source: Sci Rep. 2019 Jan 30;9:942. doi: 10.1038/s41598-018-37806-x (PMC6353924; doi:10.1038/s41598-018-37806-x)
Supplement: Supplementary file 1 — Dataset 1 [file 41598_2018_37806_MOESM1_ESM.pdf]

Supplementary Information

Creation of haemoglobin A1c direct oxidase from fructosyl peptide oxidase by combined structure-based site specific mutagenesis and random mutagenesis

Noriyuki Ogawa[1], Takehide Kimura[1], Fumi Umehara[1], Yuki Katayama[1], Go Nagai[1], Keiko Suzuki[2], Kazuo Aisaka[2†], Yukie Maruyama[3,4], Takafumi Itoh[3,5], Wataru Hashimoto[3], Kousaku Murata[3,4], & Michio Ichimura[1]

[1]Research Laboratories, Kyowa Medex Co., Ltd., 600-1, Minami-isshiki, Nagaizumi-cho, Sunto-gun, Shizuoka, 411-0932, Japan.

[2]R&D Division, Kyowa Hakko Kirin Co., Ltd., 3-6-6, Asahi-machi, Machida-shi, Tokyo, 194-8533, Japan.

[3]Division of Food Science and Biotechnology, Graduate School of Agriculture, Kyoto University, Uji, Kyoto 611-011, Japan.

[4]Department of Life Science, Faculty of Science and Engineering, Setsunan University, Neyagawa, Osaka 572-8508, Japan.

[5]Faculty of Biotechnology, Fukui Prefectural University, 4-1-1 Kenjojima, Matsuoka, Eiheiji-cho, Yoshida-gun, Fukui, 911-1195, Japan.

†Deceased on 16th November 2010.

Correspondence and requests for materials should be addressed to N. O. (email: noriyuki-ogawa@kyowa-kirin.co.jp)

Results

Obtaining the template enzyme for modification

In the course of screening our fugal library, fructosyl peptide oxidase (AnFPOX-1) of *Aspergillus nidulans* KH125 was selected because of its highest reactivity ratio for α-fructosyl valine (F-V) over ε-fructosyl lysine (ε-F-K) (F-V/ε-F-K signal ratio: 28.4). The amino acid sequence of wild -type AnFPOX-1 shared high identity with those of known group I FPOX species including *Eupenicillium terrenum* FPOX (89%)[1] and *Phaeosphaeria nodorum* FPOX (83%)[2].

The recombinant protein of AnFPOX-1 was successfully prepared using the *Escherichia coli* DH5α strain harbouring the expression plasmid pTrc-AnFPOX-1. AnFPOX-1 exhibited highly specific oxidation activity for F-V (0.557 U/mg) and weak reactivity for ε-F-K (0.012 U/mg), supporting the reported group I FPOX nature of AnFPOX-1[3]. Conversely, AnFPOX-1 displayed no reactivity for fructosyl valyl histidine (F-VH), a target fructosyl substance in the current HbA1c enzymatic assay.

Improving enzymatic characteristics via random mutagenesis

The enzymatic specification of AnFPOX-1 was improved using oxidation activity for F-VH and thermal stability as indices for modification via random mutagenesis. AnFPOX-2, generated by introducing an S71Y mutation in AnFPOX-1, exhibited significant F-VH oxidation activity for the first time. After subsequent rounds of random mutagenesis using the pTrc-AnFPOX-2 plasmid as a template, AnFPOX-15 was generated by introducing an additional ten mutations. The F-VH oxidation activity of AnFPOX-15 was improved by 7.5-fold compared with that of AnFPOX-2 (Table **S1**). No mutants generated in these trials exhibited reactivity for fructosyl peptides longer than F-VH and, let alone for whole HbA1c molecule.

Concurrently, the thermal stability of the generated mutants was evaluated by measuring the residual activity after heat treatment at 55°C for 15 min. The thermal stability of AnFPOX-15 was improved by 17.4-fold compared with that of AnFPOX-1 (Table **S1**).

Methods

Activity-based screening using a fungal library

Fructosyl substrates (F-V, F-VH and ε-F-K) were synthesised and purchased from Peptide

Institute, Inc. (Osaka, Japan)

Our fungal library (122 strains) was subjected to enzyme screening by evaluating the reactivity ratio for fructosyl substrates (F-V/ε-F-K). Each strain was inoculated in YPD liquid medium and cultured with shaking at 30°C and 200 rpm for 2 days. Grown mycelia were collected via filtration and homogenised after freezing using liquid nitrogen. By adding 10 mM potassium phosphate buffer (KPB, pH 7.0) containing protease inhibitor cocktail, internal proteins were extracted as the samples for reactivity evaluation.

For the evaluation of reactivity, a colorimetric method using oxidase and peroxidase (POD) was employed. A 190 μL mixture containing of 10 mM KPB (pH7.0), 3.5 U/L POD, 0.5 mM 4-aminoantipyrine, 0.5 mM N-ethyl-N-(2-hydroxy-3-sulfopropyl)-3-methylaniline and 20 μL of 1 mM substrate (F-V or ε-F-K, final concentration of 0.09 mM) was mixed in a multi-well plate. Then, 10 μL of the enzyme samples were added to initiate the reaction. The absorbance change at 550 and 690 nm (reference) during incubation for 10 min at 30°C was measured using an Infinite F200 (Tecan, Männedorf, Switzerland). Signal intensities detected using F-V and ε-F-K were compared, and the strain that exhibited a high F-V/ε-F-K ratio was selected as a candidate.

Preparing recombinant enzyme

Aspergillus nidulans (KY125) was cultured on potato dextrose agar medium, and the genomic DNA of the cultured mycelia was prepared using NucleoSpin Plant II (TaKaRa Bio, Japan). The hypothetical FPOX sequence of *A. nidulans* genomic DNA was identified via a Blast search against a DNA database (http://www.aspgd.org/) using the cDNA sequence of fungal FPOX (BAD00186.1| fructosyl peptide oxidase [*Coniochaeta sp.* NISL 9330]) as a query. Referring to this sequence, the primer pair of AnFPOX-F (5′-AATGCCATG**G**CGCCCCGAGCCAACACCAAAATC-3′, underline denotes an Nco I site and bold indicates an artificial base replacement to introduce an Nco I site) and AnFPOX-R primer (5′-AATGGGATCCCTACATCTTTGCCTCATTCCTCCAC-3′, underline denotes a Bam HI site) was designed, reflecting the 5' or 3' sequence of the hypothetical FPOX sequence. Using these primers, the genomic DNA sequence of the putative FPOX was amplified using KOD-plus DNA polymerase (Toyobo, Japan) following the manufacturer’s protocol. The PCR fragment was purified and digested by Nco I and Bam HI endonucleases to introduce the digested product into the multi-cloning site of the pTrc99A vector (Amersham Pharmacia). After sequence analysis, the

determined sequence and that in the database were compared to identify intron sequences. The cDNA sequence of FPOX was constructed via iterative overlap extension PCR procedures[4] using the primer pairs shown in Table **S2**. The cDNA fragment was introduced into the pTrc99A vector to construct the expression plasmid, pTrc-FPOX-1. *E. coli* DH5α competent cells were transformed by pTrc-FPOX-1 to develop a recombinant FPOX expression system.

An *E. coli* strain harbouring an expression vector was inoculated into 5 mL of Luria-Bertani (LB) liquid medium (50 mg/L Amp.) and cultured with shaking at 37 °C overnight. Subsequently, 2 mL of the culture were inoculated into 200 mL of fresh LB medium (50 mg/L Amp.). After subsequent culturing with shaking at 37 °C for 2 h, 100 mM IPTG solution was added to induce protein expression (final concentration of 0.1 mM).

Cultured cells were collected via centrifugation, suspended in 10 mM KPB (pH 7.0) and disrupted by sonication. The homogenate was centrifuged and filtrated using a 0.22 μm membrane to prepare a cell-free extract. The obtained cell-free extract was subjected to ammonium sulphate precipitation at 60% saturation to afford a pellet, and the pellet was dissolved in 10 mM KPB (pH 7.0). The obtained solution was then subjected to a butyl-Sepharose column equilibrated with 10 mM KPB (pH 7.0) and 1 M $(NH_4)_2SO_4$. After washing with the same buffer, the FPOX fraction was eluted isocratically with 10 mM KPB (pH 7.0) and 0.5 M $(NH_4)_2SO_4$. The FPOX fraction was then dialysed against 10 mM KPB (pH 7.0) overnight at 4°C. The dialysed sample was subjected to a DEAE-Toyopearl column equilibrated with 10 mM KPB (pH 7.0), and the flow-through fraction was collected.

The purity of the sample was confirmed via SDS-PAGE. Protein contents were determined by measuring the absorbance at 280 nm using a U-3000 spectrophotometer and assuming that E_{280} = 1.48 (AnFPOX-1) corresponded to 1 mg/mL[5]. The contents of other mutants were also determined in the same manner using each E_{280} values.

Random mutagenesis via error-prone PCR

Random mutagenesis of AnFPOX-1 was conducted using a Gene Morph II Random Mutagenesis Kit (Stratagene, San Diego, CA, USA) following the manufacturer's protocol using the primers AnFPOX-F and AnFPOX-R. In total, 300 ng of the template plasmid in 50 μL of the PCR mix achieved the desired error rate (1 base error/kb). Amplified PCR fragments were purified and introduced into a multi-cloning site of the

expression vector. *E. coli* DH5α was transformed by the expression vector and cultured in LB agar plate medium (50 mg/L Amp.). Grown colonies were used for evaluation as a random mutagenesis library. Approximately 5000 clones were used to evaluate the enzymatic characteristics following the aforementioned method.

Activity assay of generated mutant enzymes

The oxidation activity for F-VH (F-VH oxidation activity) was evaluated using the same method employed for screening the fungal library. Oxidation activity was calculated via the generation of hydrogen peroxide using the calibration curve prepared from measurements using samples with known concentrations of hydrogen peroxide. One unit of activity was defined as the quantity of enzyme necessary to catalyse the formation of 1 μmol of hydrogen peroxide per minute. Specific activity (U/mg) was defined as the activity catalysed by 1 mg of protein.

Activity-based screening of AnFPOX mutants

Evaluation samples on screening were prepared as follows. For pre-culture, each transformant was inoculated in 200 μL of LB medium (50 mg/L Amp.) and cultured with shaking overnight at 37 °C. Two microlitres of culture were transferred to 200 μL of fresh LB medium in a multi-well plate and subjected to culturing. After an additional 2 h, 100 mM IPTG solution was added to induce protein expression (final concentration of 0.1 mM), followed by culturing with shaking for 12 h at 37 °C. Cultured cells were collected by centrifugation and then lysed using 50 μL of BugBuster solution (Merck Millipore, MA, USA). The supernatant of lysed cells was subjected to an activity assay using 1 mM F-VH and ε-F-K as the substrates (final concentration of 0.09 mM) following the previously described method.

Evaluation of the thermal stability of mutant enzymes

The cell-free extract of FPOX-expressing *E. coli* or purified enzymes was subjected to heat treatment using the Gene Amp PCR System 9700 (Applied Biosystems, Foster City, CA, USA) for 10 min at 50 °C (for crude extract on screening) or 15 min at 55 °C (for purified enzymes on evaluation of enzymatic specification). Residual activity was calculated from the signal intensity after heat treatment comparing with that for non-treated samples using F-V as the substrate.

Table S1 Summary of fructosyl valyl histidine (F-VH) reactivity and thermal stability of the AnFPOX mutants

*Bold indicates mutations newly introduced in the mutant.

**Specific activity (U/mg) is presented as fold changes relative to that of AnFPOX-2, which was set as 1.0.

Mutant	Mutation (number of mutation)*	F-VH reactivity		Thermal stability	
		Specific activity (U/mg)	Fold**	Residual activity (%)	Fold
AnFPOX-1	none (0)	n.d.	-	4.92 ± 0.61	1.0
AnFPOX-2	**S71Y** (1)	0.019 ± 0.001	1.0	16.0 ± 1.20	3.3
AnFPOX-3	S71Y/**K109R** (2)	0.048 ± 0.002	2.5	2.20 ± 0.80	0.4
AnFPOX-7	S71Y/K109R/**I94M/F269I** (4)	0.074 ± 0.001	3.9	38.8 ± 4.40	7.9
AnFPOX-10	S71Y/K109R/**I94M/F269I/E104K/S59G** (6)	0.106 ± 0.003	5.6	38.9 ± 2.73	7.9
AnFPOX-15	S71Y/K109R/I94M/F269I/E104K/S59G/**M58F/G105K/G183D/P302L/N272D** (11)	0.142 ± 0.003	7.5	85.1 ± 8.59	17.4

Table S2. Primer sequences used for overlap extension PCR for constructing the cDNA sequence of AnFPOX-1

Primer	Sequence (5′-3′)	Target
AnFPOX-F	AATGCCATGGCGCCCCGAGCCAACACCAAAATC (Underline: Nco I site)	Exon1
Ex1-R	CTTCCTCTGTTGAAGAGACGTCGATCATTCCAACATTGTGGAAAAAC	
Ex2-F	CACGCAGGAGCAGATTAAAGGCTGGAAAGGCCTGTTCTGTGGCGA	Exon2
Ex2-R	CCACAGAACAGGCCTTTCCAGCCTTTAATCTGCTCCTGCGTGAAATG	
Ex3-F	CACGCAGGAGCAGATTAAAGGCTGGAAAGGCCTGTTCTGTGGCGAC	Exon3
Ex3-R	GGCTTTTTGAACGTGCCGGCGCCAGCCGCCGTCGCCACAGAACAGG	
Ex4-F	CGTCAAGTTTGGATTCGGCGGCGCCGGCACGTTCAAAAAGCCACTCTTC	Exon4
Ex4-R	GATGTGGGCAAAGACCCAGGCCTTTGAAACGCACTGCTCCTCCAG	
Ex5-F	GAGGAGCAGTGCGTTTCAAAGGCCTGGGTCTTTGCCCACATCCAACTG	Exon5
Ex5-R	CAGACTTTTATGATGCCGTTTTCATTCGGCTCAAAGAAAAACCCAT	
Ex6-F	GTTTTTCTTTGAGCCGAATGAAAACGGCATCATAAAAGTCTGTGAC	Exon6
AnFPOX-R	AATGGGATCCCTACATCTTTGCCTCATTCCTCCAC (Underline: Bam HI site)	

Table S3. FPOX of which crystal structures were determined n.d.: determined but not disclosed.

Enzyme	Origin	Amino acid	Identity (%) vs AnFPOX-15	FPOX group	PDB accession id	rmsd vs AnFPOX-15	Ref.
AnFPOX-15	*Aspergillus nidulans*<br>(Modified enzyme)	438	-	I	6A6R<br>6A6S<br>6A6T<br>6A6U<br>6A6V	-	-
AfFAOX-I	*Aspergillus fumigatus*	445	36	II	4WCT<br>4XWZ	1.77 Å<br>(385Cα)	6,7
AfFAOX-II		438	37	III	3DJD<br>3DJE	2.04 Å<br>(387Cα)	8
EtFPOX	*Eupenicillium terrenum*	437	89	I	4RSL	0.80 Å<br>(428Cα)	9
PnFPOX	*Phaeosphaeria nodorum*	437	83	I	5T1E<br>5T1F<br>5XAO	1.12 Å,<br>(424Cα)	10
CoFPOX	*Coniochaeta sp.*<br>(Modified enzyme))	437	74.5	I	n.d.	-	11

Table S4. Statistics for X-ray crystallographic data and refinement

[a] The highest resolution shell is shown in parentheses.

	SeAnFPOX-15	SeAnFPOX-15/FSA	AnFPOX-15 R61G	AnFPOX-15 R61G/FSA	AnFPOX-21/FSA
**Data collection**					
Wavelength (Å)	0.97980	1.00000	1.00000	1.00000	0.98000
Resolution range (Å)	50.0 -2.60 (2.64 -2.60) [a]	50.0 -1.85 (1.92 -1.85) [a]	50.0 -1.90 (1.93 -1.90) [a]	50.0 -1.95 (1.98 -1.95) [a]	50.0-2.90 (3.00-2.90) [a]
Space group	$P3_221$	$P3_221$	$P3_221$	$P3_221$	$P6_5$
Unit-cell parameters (Å)					
a, b	73.20	72.76	72.34	72.92	135.54
c	162.31	159.69	160.11	150.05	144.68
Total observations	322470	597730	186327	380400	319723
Unique reflections	29504	42549	38889	36930	33508
Completeness (%)	100 (100)	99.7 (97.8)	99.6 (100)	99.8 (99.5)	99.9 (99.1)
$I/\sigma(I)$	44.3 (8.3)	63.6 (5.5)	14.1 (4.7)	44.2 (8.6)	14.0 (2.3)
R_{merge}	0.090 (0.410)	0.069 (0.383)	0.057 (0.405)	0.077 (0.461)	0.130(0.852)
$CC_{1/2}$ in outer shell	0.974	0.975	0.934	0.958	0.727
**Refinement**					
Resolution range (Å)	50 -2.60	50 -1.85	50 -1.90	50 -1.95	50 -2.90
R_{work}/R_{free}	0.218/0.264	0.190/0.224	0.230/0.262	0.164/0.197	0.166/0.207
No. atoms					
Protein	3381	3381	3367	3367	6733
FAD	53	53	53	53	106
FSA	-	16	-	16	32
oxidised DTT	16	16	40	24	-
SO_4^{2-}	5	20	20	5	-
Water molecule	46	203	162	365	-
rmsd					
Bond lengths (Å)	0.009	0.012	0.010	0.010	0.009
Bond angles (deg.)	0.896	1.244	1.163	1.600	1.222
Ramachandran plot					
Most favoured (%)	96.3	97.7	97.7	97.4	96.3
Allowed (%)	3.7	2.3	2.3	2.6	3.7

Table S5. Hydrogen bonds (<3.3Å) between ligand molecules and AnFPOX-15

Ligand		AnFPOX-15/FSA		
Molecule	Atom	Residue	Atom	Distance (Å)
FAD	O4	I57	N	2.6
	N3	I57	O	2.9
	O2	K380	N	3.1
		F379	N	3.2
	O3’	S378	N	3.3
		D374	OD1	2.6
	O4’	T17	OG1	2.8
	O2P	M18	N	2.9
		T17	OG1	2.8
	O1A	T17	N	2.9
			OG1	2.9
		S49	OG1	2.7
	O2A	A50	N	2.9
	O3B	D40	OD1	2.8
		S46	OG	3.1
	O2B	D40	OD2	2.5
		S46	OG	2.9
		Q48	NE2	3.0
	N1A	F187	N	2.9
FSA	OAB	K380	NZ	2.6
	OAA	K380	NZ	3.0
		H377	ND1	2.9
	OAJ	G376	O	3.0
	OAC	G376	O	3.1
		R419	NH1	3.2
			NH2	3.2
	OAD	E282	OE1	2.7
		R419	NH1	3.0
	OAE	E282	OE2	2.9

Table S6. Primer Sequences used for creation of HbA1c direct oxidase in the report

Primer	Sequence (5′-3′)
pTrc-F	CAATTAATCATCCGGCTCGTA
pTrc-R	CTTCTGAGTT CGGCATGGGG
R61G-F	CTGAACAAAATCTTCGGCATCGGGCTGCGCAACAAGCCTGAC
R61G-R	GTCAGGCTTGTTGCGCAGCCCGATGCCGAAGATTTTGTTCAG
R61A-F	CTGAACAAAATCTTCGGCATCGCGCTGCGCAACAAGCCTGAC
R61A-R	GTCAGGCTTGTTGCGCAGCGCGATGCCGAAGATTTTGTTCAG
R61Sat-F	CCTGAACAAAATCTTCGGCATCNNSCTGCGC
R61Sat-R	GTAAGTCAGG CTTGTTGCGCAGSNNGATGCC
R63Sat-F	AAAATCTTCGGCATCGGCCTGNNSAACAAG
R63Sat-R	GAGTTGTAAGTCAGGCTTGTTSNNCAGGCC
L62Sat-F	AACAAAATCTTCGGCATCTCANNSGCGAAC
L62Sat-R	TTGTAAGTCAGGCTTGTTCGCSNNTGAGAT
Bgl II-F	CAGTCTGCAGGCTACGACCTGAACAAGATC
Bgl II-R	GATCTTGTTCAGGTCGTAGCCTGCAGACTG
Y71Sat-F	GAACAAGCCTGACTTACAACTCNNSCTTGAG
Y71Sat-R	CCACATGTCCAGCGCCTCAAGSNNGAGTTG
L75Sat-F	CTTACAACTCTCGCTTGAGGCGNNSGACATG
L75Sat-R	GAGGATCATTTTTCCACATGTCSNNCGCCTC
M108Sat-F	GAAGGCATCAAAAAGCTTCGCNNSAGATAC
M108Sat-R	GCGGAGAAGAGACTGGTATCTSNNGCGAAG
D115Sat-F	CATGAGATACCAGTCTCTTCTCNNSGCAGGC
D115Sat-R	GTCTTCTCGAGCCCAATGCCTGCSNNGAGAAG
P66Sat-F	GGCATCTCAGGCGCGAACAAGNNSGACTTA
P66Sat-R	CTCAAGGCTGAGTTGTAAGTCSNNCTTGTT
F342Sat-F	GAGGTTCAATGACAAGGAACTGNNSAACAGG
F342Sat-R	GCACCAGCACATGGCCCTGTTSNNCAGTTC
A355Sat-F	TGGTGCACCGATACCGCGGATNNSAATCTG
A355Sat-R	GATGCTCACAAACAAGCAGATTSNNATCCGC
EP1-R	CCAGCAGGAAATTCGTCTTCTCGAG
EP2-R	CCAGCAGGAAATTCGTCTTCTCGAG
EP3-F	GTCACCATCAAAAAGGCTATCAAC

N: A, T, G, or C, S: G or C.

Figure S1. Amino acid sequence alignment of group I FPOX and AfFAOX-II (group III)

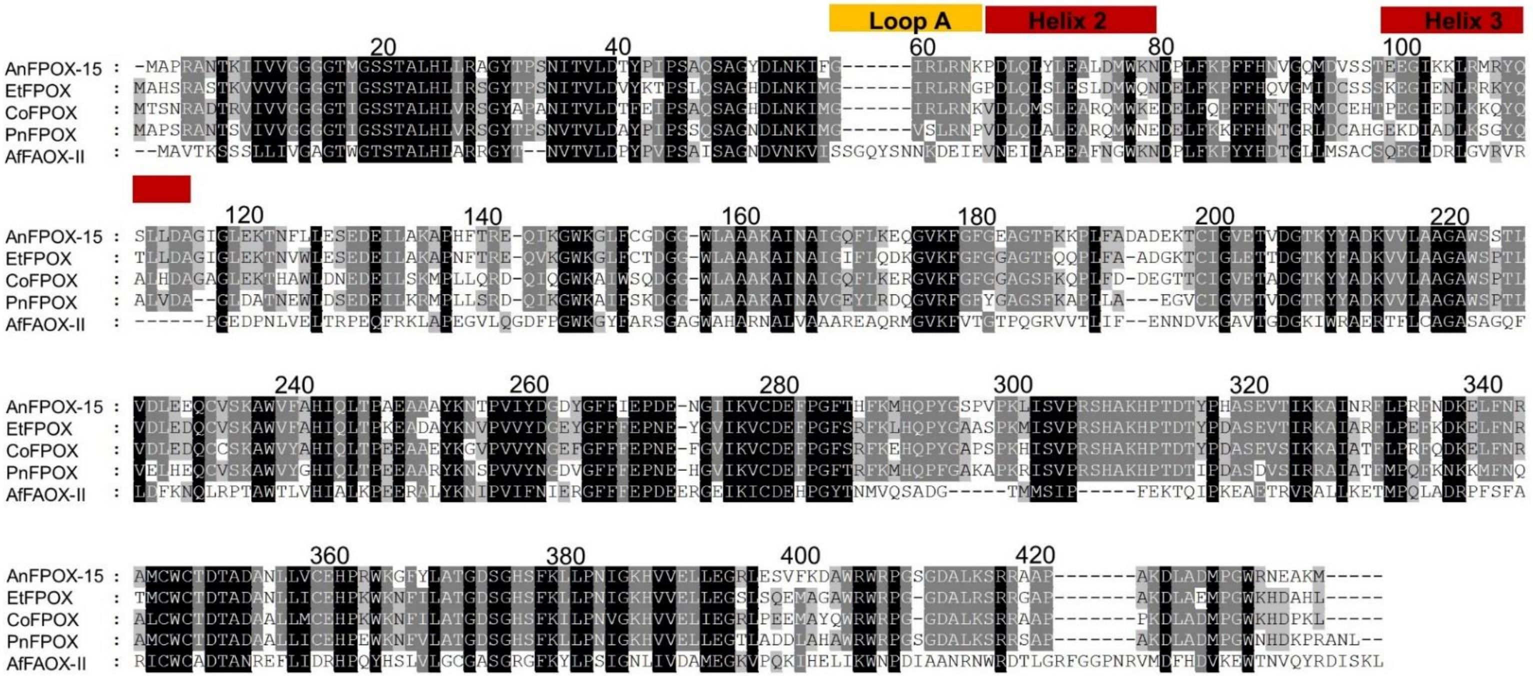

Amino acid sequences of AnFPOX-15, EtFPOX (BAD00185.1 fructosyl peptide oxidase [*Penicillium terrenum*]), CoFPOX (BAD00186.1| fructosyl peptide oxidase [*Coniochaeta sp.* NISL 9330]), PnFPOX (XP_001798711.1| hypothetical protein SNOG_08398 [*Phaeosphaeria nodorum* SN15 and AfFAOX-II (AAC49711.1 fructosyl amine: oxygen oxidoreductase [*Aspergillus fumigatus*]) were aligned using clustalW programme (https://www.genome.jp/tools-bin/clustalw). Sequence number was assigned based on AnFPOX-15 sequence. Highly conserved residues were shown as black box and white term. Sequences corresponding to Loop A, helix 2 and helix 3 of AnFPOX-15 were shown by coloured bar.

Figure S2. Random mutagenesis via error-prone PCR

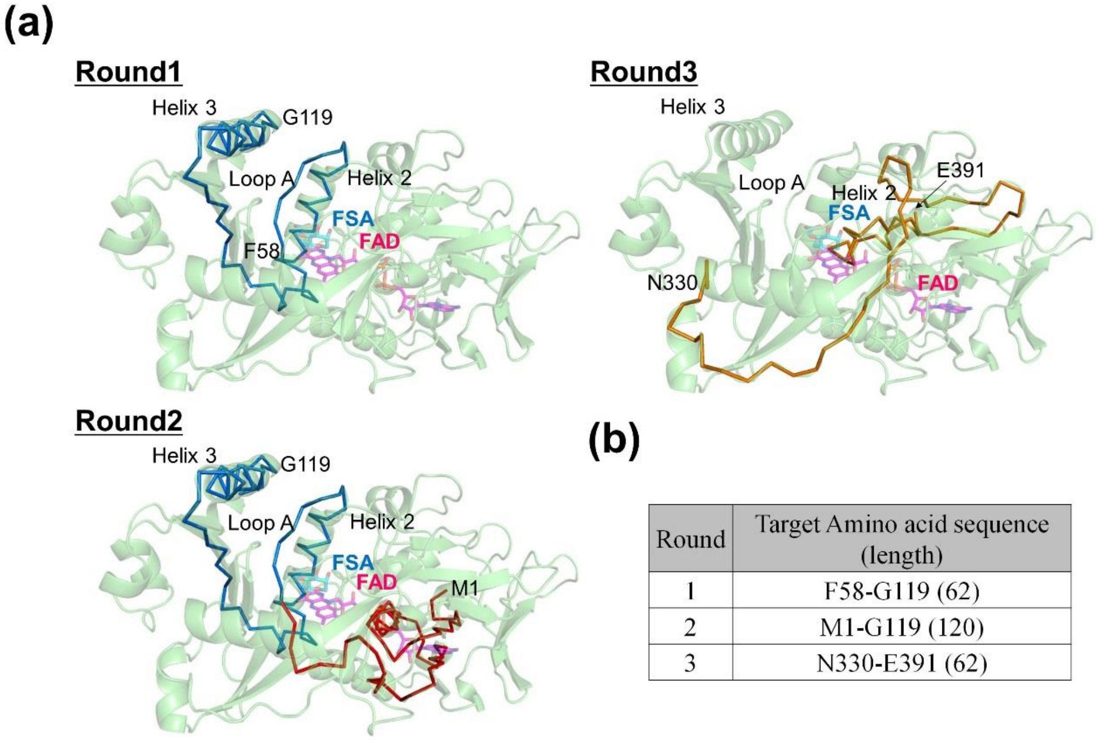

(b)

Round	Target Amino acid sequence (length)
1	F58-G119 (62)
2	M1-G119 (120)
3	N330-E391 (62)

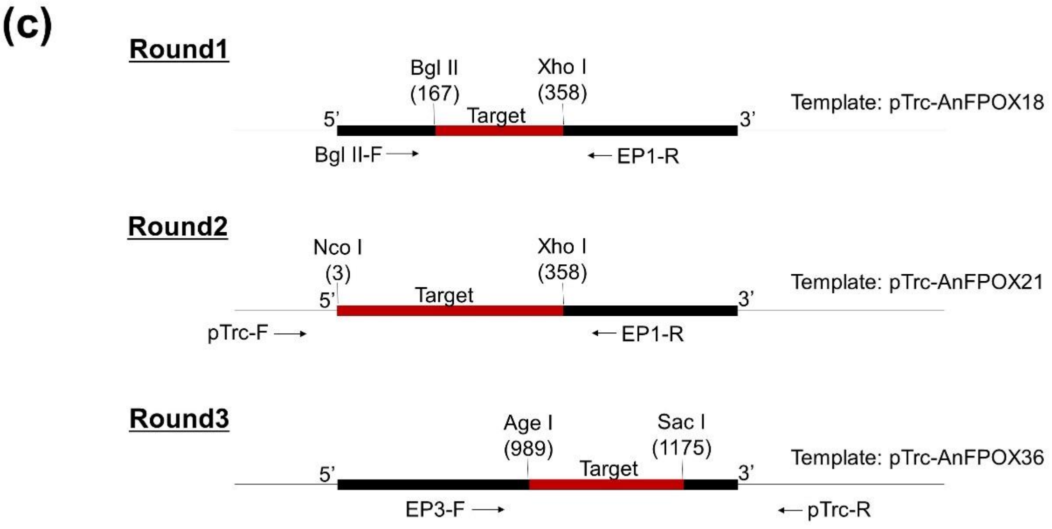

(a) Target regions of random mutagenesis illustrated on AnFPOX-15/FSA structure. (b) Separated target regions for each round. (c) Target amino acid region and number. (d) Methodology of amplification of PCR fragment including errors by error prone PCR. Black bold bar indicate AnFPOX on pTrc-AnFPOX plasmid, and red bar indicates a target region for the round.

Black arrows indicate the primer pairs used for the amplification of target fragment.

Figure S3. Thermal stability of AnFPOX mutants

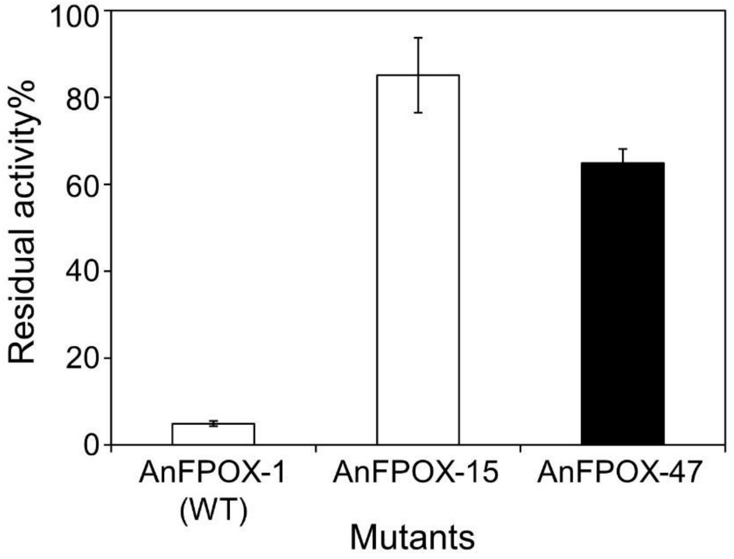

Thermal stability of generated mutants were evaluated by measuring the residual reactivity after heat treatment at 55°C for 15 min using F-V as a substrate. Data are mean ± SD (n =3).

Figure S4. Structural model of P66 mutation in AnFPOX-21/FSA

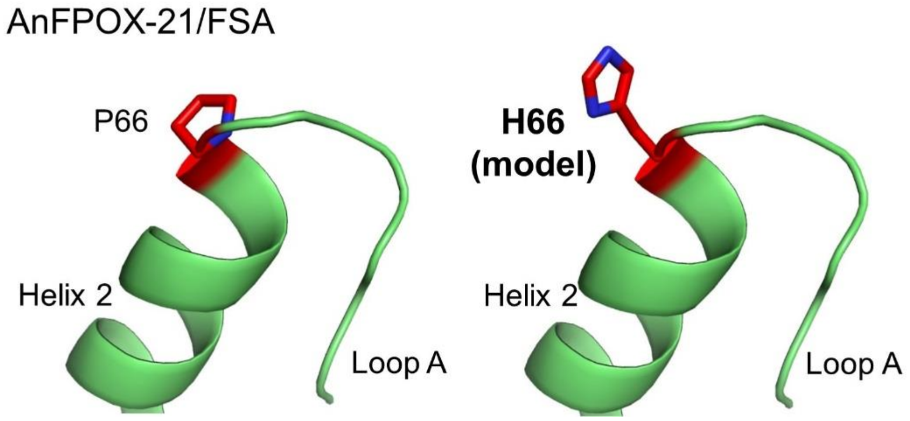

Loop A and helix 2 of AnFPOX-21/FSA crystal structure and that of AnFPOX-21/FSA P66H model structure generated by PyMOL programme.

Figure S5. Location of F342 residue and replacing effect on reactivity

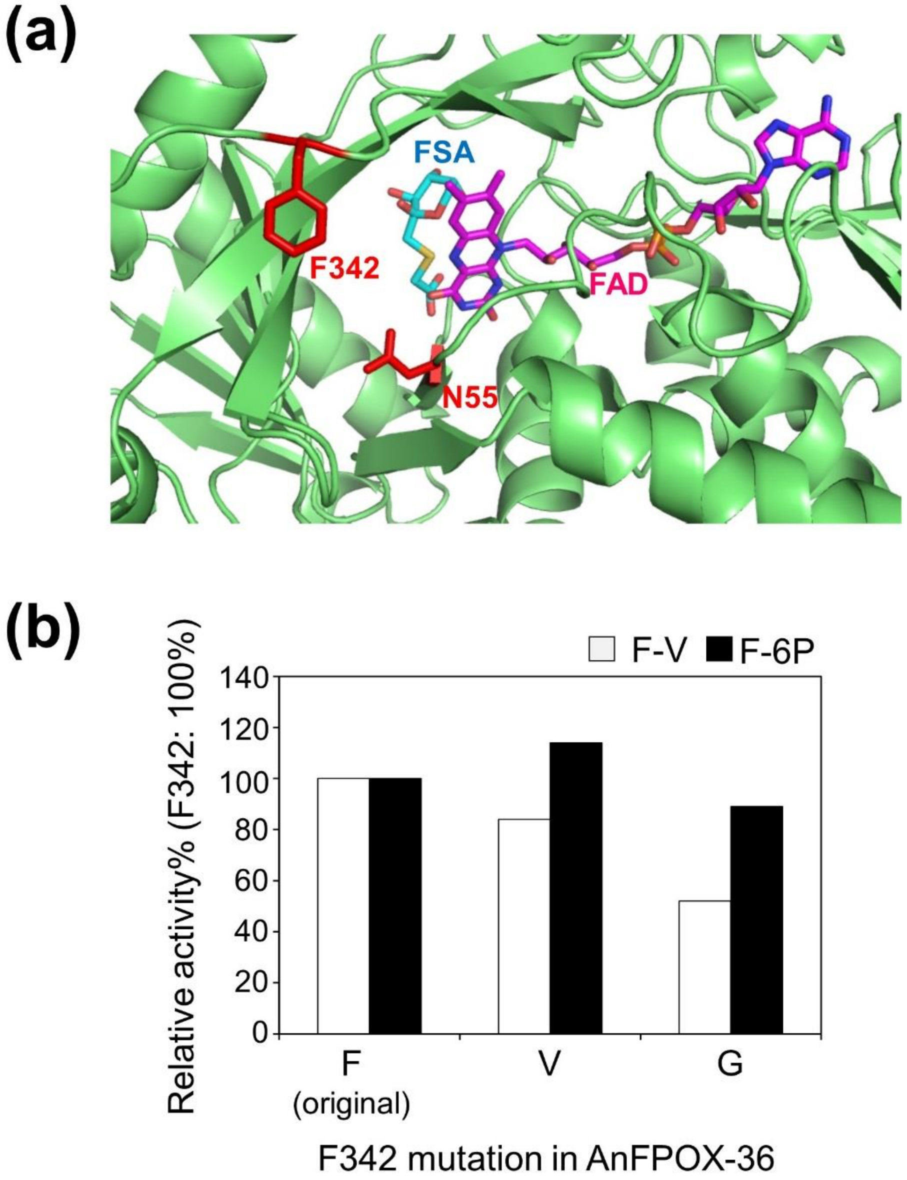

(a) Looking up view from *si*-face of FAD contained in AnFPOX-21/FSA. F342 and N55 (corresponding residue with N56 of PnFPOX) are shown as a stick in red. The figure was generated by PyMOL programme. (b) F342 mutation effect in AnFPOX-36 on reactivity for F-V and F-6P. Data was shown as relative activity% based on the value of F342 (original) as 100%.

Figure S6. Location of A355 residue in the AnFPOX-21/FSA structure

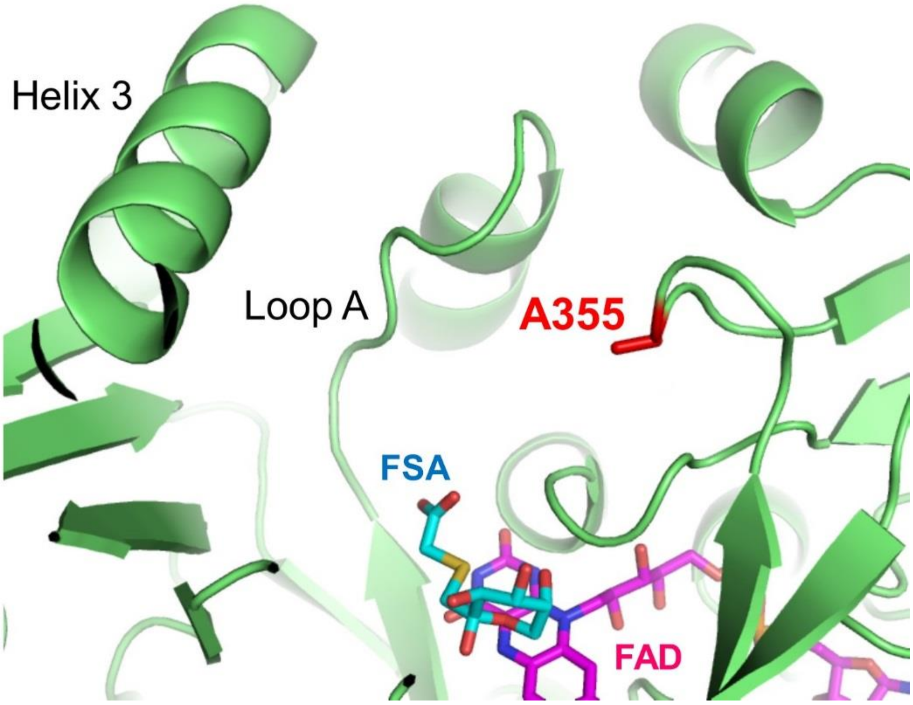

The active site of AnFPOX-21/FSA structure was shown as cartoon model. A355 residue was shown as a stick in red. FSA and FAD are shown in cyan and magenta respectively in stick models. The figure was generated by PyMOL programme.

Figure S7. SDS-PAGE analysis of prepared Apo haemoglobin (ApoHb) and non-treated haemoglobin (Hb)

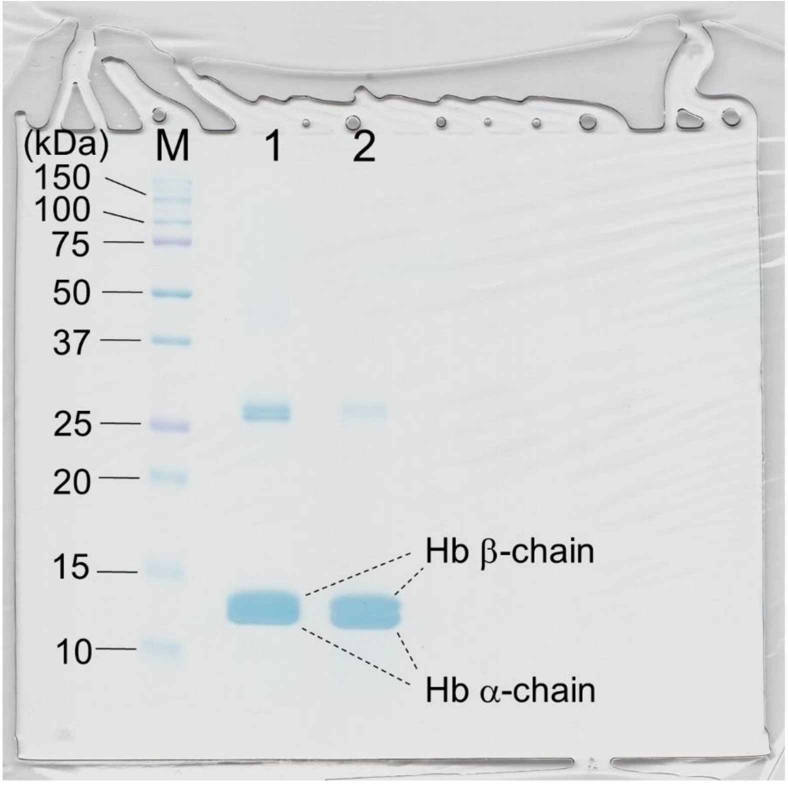

Lane M, molecular weight marker; Lane 1, ApoHb; Lane 2, Hb (non-treated). Each 5 μg of protein was subjected under reduced and degenerated condition using 15% polyacrylamide gel.

Figure S8. SDS-PAGE analysis of each of the purified AnFPOX mutants

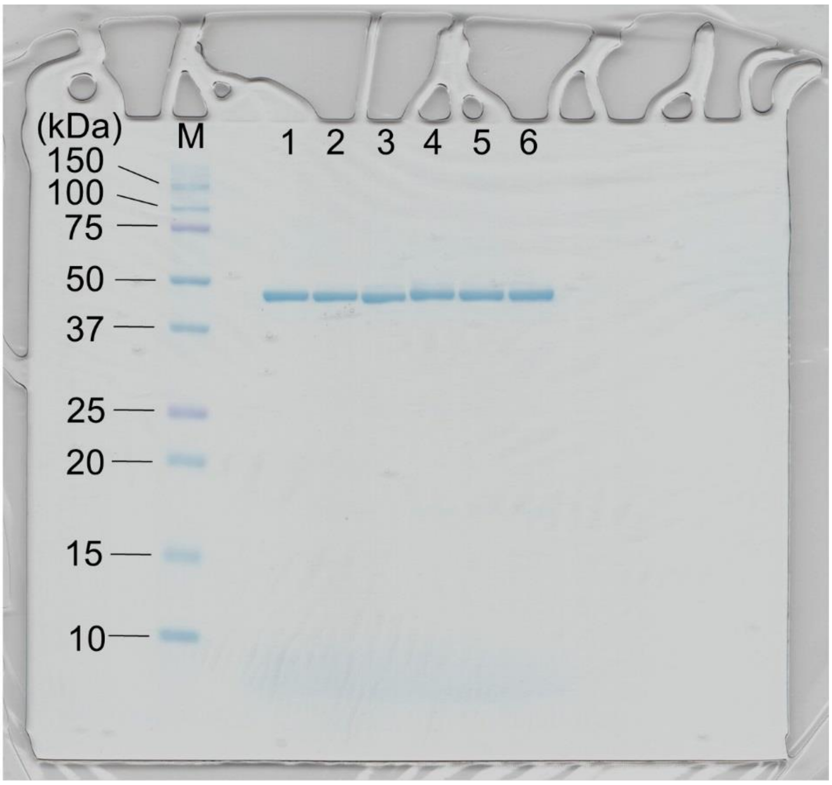

Lane M: Molecular weight markers. Each of the lanes (1-6) contains each of 3 μg of the purified AnFPOX mutants. Electrophoresis was performed using 15% polyacrylamide gel under reduced, degenerated condition.
Lane 1, AnFPOX-15; Lane 2, AnFPOX-15 R61S; Lane 3, AnFPOX-18; Lane 4, AnFPOX-21; Lane 5, AnFPOX-36; Lane 6, AnFPOX-47. The electrophoresis pattern of each of the mutants was correspondent with expected molecular weight (approximately 48 kDa) calculated from amino acid sequence.

Reference

1 Hirokawa, K., Gomi, K. & Kajiyama, N. Molecular cloning and expression of novel fructosyl peptide oxidases and their application for the measurement of glycated protein. *Biochemical and biophysical research communications* **311**, 104-111 (2003).

2 Kim, S., Ferri, S., Tsugawa, W., Mori, K. & Sode, K. Motif-based search for a novel fructosyl peptide oxidase from genome databases. *Biotechnology and bioengineering* **106**, 358-366, doi:10.1002/bit.22710 (2010).

3 Ferri, S., Kim, S., Tsugawa, W. & Sode, K. Review of fructosyl amino acid oxidase engineering research: a glimpse into the future of hemoglobin A1c biosensing. *Journal of diabetes science and technology* **3**, 585-592, doi:10.1177/193229680900300324 (2009).

4 Higuchi, R., Krummel, B. & Saiki, R. K. A general method of in vitro preparation and specific mutagenesis of DNA fragments: study of protein and DNA interactions. *Nucleic acids research* **16**, 7351-7367 (1988).

5 Gill, S. C. & von Hippel, P. H. Calculation of protein extinction coefficients from amino acid sequence data. *Analytical biochemistry* **182**, 319-326 (1989).

6 Rigoldi, F. *et al.* Molecular dynamics simulations provide insights into the substrate specificity of FAOX family members. *Molecular bioSystems* **12**, 2622-2633, doi:10.1039/c6mb00405a (2016).

7 Rigoldi, F. *et al.* Crystal structure of the deglycating enzyme Amadoriase I in its free form and substrate-bound complex. *Proteins* **84**, 744-758, doi:10.1002/prot.25015 (2016).

8 Collard, F. *et al.* Crystal structure of the deglycating enzyme fructosamine oxidase (amadoriase II). *The Journal of biological chemistry* **283**, 27007-27016, doi:10.1074/jbc.M804885200 (2008).

9 Gan, W. *et al.* Structural basis of the substrate specificity of the FPOD/FAOD family revealed by fructosyl peptide oxidase from *Eupenicillium terrenum*. *Acta crystallographica. Section F, Structural biology communications* **71**, 381-387, doi:10.1107/s2053230x15003921 (2015).

10 Shimasaki, T., Yoshida, H., Kamitori, S. & Sode, K. X-ray structures of fructosyl peptide oxidases revealing residues responsible for gating oxygen access in the oxidative half reaction. *Scientific reports* **7**, 2790, doi:10.1038/s41598-017-02657-5 (2017).

11 Ichiyanagi, A. *et al.* Crystallization and preliminary crystallographic analysis of two eukaryotic fructosyl peptide oxidases. *Acta crystallographica. Section F, Structural biology and crystallization communications* **69**, 130-133, doi:10.1107/s1744309112051445 (2013).
